# Perspectives on conducting “sex-normalising” intersex surgeries conducted in infancy: A systematic review

**DOI:** 10.1371/journal.pgph.0003568

**Published:** 2024-08-28

**Authors:** Luke Muschialli, Connor Luke Allen, Evelyn Boy-Mena, Aiysha Malik, Christina Pallitto, Åsa Nihlén, Lianne Gonsalves

**Affiliations:** 1 Department of Public Health and Primary Care, University of Cambridge, Cambridge, United Kingdom; 2 Faculty of Medicine, Nursing and Health Sciences, Monash University, Melbourne, Australia; 3 Department of Gender, Rights and Equity, World Health Organization, Geneva, Switzerland; 4 Department of Mental Health and Substance Use, World Health Organization, Geneva, Switzerland; 5 Department of Sexual and Reproductive Health and Research, UNDP-UNFPA-UNICEF-WHO-World Bank Special Programme of Research, Development and Research Training in Human Reproduction (HRP), World Health Organization, Geneva, Switzerland; Centre of Biomedical Ethics and Culture, PAKISTAN

## Abstract

Children with intersex variations continue to be subject to elective, irreversible, “sex-normalising” surgical interventions, despite multiple human rights and legislative bodies calling for their prohibition. Our systematic review aims to understand how medical literature reports rationales for “sex-normalising” surgical interventions conducted in childhood, and how they are contextualised within the medical and social controversy surrounding such interventions. PubMed, EMBASE and CINAHL were searched for English language, peer-reviewed articles reporting primary data on elective, genital, “sex-normalising” surgical interventions conducted on individuals <10 years, published 01/07/2006-30/06/2023 (PROSPERO ID: CRD42023460871). Data on outcomes reported, rationale for the conduct and timing of interventions and acknowledgement of controversy were extracted. Narrative synthesis described rationales and controversy. Risk of bias was assessed using Johanna Briggs Institute Tools. 11,042 records were retrieved, with 71 articles included for analysis. One of the most common outcomes collected in included literature were cosmetic outcomes, primarily reported by surgeons or parents. 62.0% of studies reported no rationale for intervention timing, 39.4% reported no rationale for conduct and 52.1% acknowledged no controversy in intervention conduct. Rationales included parental desire for intervention, anatomical/functional/cosmetic reasons, and a perceived goal of aligning with sex assigned by surgical teams or parents. Controversies addressed included concerns about the quality of interventions, the ethics of intervention conduct and gendered and social considerations. “Sex-normalising” interventions are conducted based largely on rationales that were not adequately supported by evidence, a desire from parents and surgeons to match genital cosmesis typically ascribed to male and female bodies, and a parental desire for intervention conduct. Legislating and medical regulatory bodies should advocate for ending the conduct of irreversible, elective, “sex-normalising” interventions conducted without the full, free and informed consent of the person concerned, to promote and protect the highest attainable standard of health for people with intersex variations.

## Introduction

Intersex is an umbrella term used to refer to individuals born with physical or biological sex characteristics (including sexual anatomy, reproductive organs and/or chromosomal patterns) that do not fit normative definitions of male or female bodies [1]. These congenital variations in sex characteristics, also known as differences/disorders of sex development (DSDs), are a large, heterogeneous group of reproductive, urogenital, chromosomal and/or hormonal congenital conditions, with the set of diagnoses lying within these umbrella terms varying across different settings and disciplines [2, 3]. Some of these variations may be visible and identifiable at birth, while others may not be recognized until later in childhood, puberty and/or adulthood [1].

Many congenital variations in sex characteristics pose no threat to physical health, while others may necessitate medical or surgical intervention (i.e., to facilitate excretion or urination). However, standard surgical practice for decades has involved using a variety of elective (i.e., non-urgent) surgical procedures to attempt to ‘normalize’ ‘atypical’ traits in people with congenital variations in sex characteristics, striving for cosmetic, functional and anatomical outcomes that align more with those associated with ‘typical’ male or female bodies [4], referred to in this manuscript as “sex-normalising” interventions. These procedures frequently take place in infancy or early childhood, due in part to pervasive beliefs that early intervention will facilitate better psychological and/or physiological development, and alleviate anticipated personal and parental stress associated with the congenital variation in sex characteristics [5–8]. Treatment options may also involve hormonal therapy, mechanical procedures (e.g., neovaginal dilations), or surgical, hormonal, and mechanical interventions which take place in adolescence, all of which are outside the scope of this review.

The continued conduct of “sex-normalising” surgical interventions in infancy or early childhood is supported by clinical guidance such as the *2006 Consensus Statement on Management of Intersex Disorders*, a statement that attempted to create recommendations for the long-term management, evaluation of, and future research into, congenital variations in sex characteristics [9]. This is despite a noted lack of evidence regarding the physical, mental, and social outcomes of infants with congenital variations in sex characteristics who have undergone such interventions, with an extant evidence-base primarily consisting of short case reports and cohort data with small sample sizes [2, 4].

Evidence on patient preference for infant surgical intervention, despite still being used as a rationale for procedures, is also inconsistent. Although some patients retrospectively express a clear preference for, and satisfaction with, early surgical intervention [10–12], others who have undergone these surgeries share dissatisfaction with long-term outcomes, citing resultant distress and trauma associated with subsequent gender identity, compromised sexual function and pleasure, dissatisfaction with genital appearance and a reflective distress surrounding their compromised autonomy for interventions practiced before they were able to articulate consent for the procedure [13–23].

Human rights experts have recently raised serious concerns about the conduct of elective, irreversible, “sex-normalising” surgical interventions carried out in infancy and childhood. In October 2016, multiple UN human rights monitoring and accountability mechanisms (The international human rights treaty bodies and special procedures behind this joint statement were the UN Committee on the Rights of the Child, UN Committee against Torture, UN Committee on the Rights of People with Disabilities, UN Sub-Committee on Prevention of Torture and other Cruel, Inhuman or Degrading Treatment or Punishment, UN Special Rapporteur on torture and other cruel, inhuman or degrading treatment or punishment, UN Special Rapporteur on the right of everyone to the highest attainable standard of health, UN Special Rapporteur on violence against women, its causes and consequences, Special Representative of the UN Secretary-General on Violence against Children, African Commission on Human and Peoples’ Rights, Council of Europe Commissioner for Human Rights and the Inter-American Commission on Human Rights.) issued a joint statement highlighting the human rights violations associated with ‘medically unnecessary surgeries […] in an attempt to forcibly change [intersex infants’, children’s and adolescents’] appearance to be in line with societal expectations about female and male bodies’ and called on governments to ’prohibit harmful medical practices on intersex children, including unnecessary surgery and treatment without their informed consent’ [24]. Following this, several UN human rights treaty bodies, including the UN Committee on the Rights of the Child [25], as well as the World Health Organization (WHO) [26], have provided further advice, speaking out against medically unnecessary surgical interventions for infants with congenital variations in sex characteristics on the grounds of compromised bodily integrity and rights of the child. Recently, and for the first time, the UN Human Rights Council adopted a resolution calling on countries to enhance efforts to combat discrimination, violence and harmful practices against people with congenital variations in sex characteristics [27]. Simultaneously, intersex community groups have extensively campaigned against “sex-normalising” interventions on the same grounds, with some suggesting that the existence of such interventions also represent an elimination of intersex communities and an enforcement of strongly normative conceptualizations of gender and sex [28]. The collective impact of advocacy groups and human rights monitoring bodies has been reflected in a growing wave of national and sub-national legislative change restricting the practice of elective genital surgery conducted on children, focusing on the protection of children’s physical integrity and the prohibition of surgical intervention for infants and children with congenital variations in sex characteristics too young to participate in decision-making [29].

Both a 2016 *Global Disorders of Sex Development Update since 2006* publication and a 2018 European publication, *Caring for Individuals with a Difference of Sex Development*, emphasise the need for clinicians to consider the ethical implications of conducting “sex-normalising” surgical interventions, and discuss the postponing of genital surgery until an individual is old enough to understand the intervention and participate in decision-making [2, 4]. Despite this, there is little evidence to suggest that the prevalence of, or rationales for conducting, “sex-normalising” surgical interventions on individuals too young to consent has changed in recent years [30, 31]. Understanding the rationale behind the continued practice of “sex-normalising” surgical interventions and how clinicians acknowledge and contextualise the controversial nature of the interventions they are practicing is necessary for identifying misconceptions in practice, and designing policy and interventions that target common drivers of continued, early surgical procedures.

Our review aims to understand the extent to which the extant medical literature is reporting rationales for elective, “sex-normalising” surgical interventions conducted in infancy and childhood, what these rationales are, and how they are contextualised within the social, medical, and political controversy surrounding “sex-normalising” interventions for people with congenital variations in sex characteristics.

## Methods

### Search and screening

Our review was compliant with PRISMA Reporting Guidelines [32] (see *S1 text*), and is registered with PROSPERO (CRD42023460871).

On 30/06/2023, PubMed, EMBASE and CINAHL were searched for relevant articles published between 01/07/2006-30/06/2023, using search terms derived from previous reviews, recommendations from field experts and terminology from the International Classification of Diseases, Version 11 (ICD-11). The search start date aligns with the year of publication of the 2006 *Consensus Statement on Management of Intersex Disorders* [9].

*S2 Text* reports our search strategy. One search filter captured medical and public health terms that covered intersex as an umbrella term as well as individual congenital variations in sex characteristics that are generally identified in infancy or early childhood (as opposed to during puberty or adulthood). A second search filter captured general surgical terms as well as specific procedures associated with genital surgery for persons with congenital variations in sex characteristics [4]. English language restrictions were applied due to the risks of losing the complex nuances of surgical rationale in translation. A final search filter attempted to exclude: animal studies; commentaries, letters, and editorials; studies covering gender-affirming care surgeries; studies focusing on adolescence or young people over the age of 10 years.

Title/abstract and full-text screening was carried out by LM and CLA. LG independently screened a random sample of 10% of full texts to ensure consensus between reviewers. Screening was conducted in the systematic review management software, *Covidence* [33].

### Inclusion and exclusion criteria

Peer-reviewed journal articles reporting primary data on elective, genital, “sex-normalising” surgical interventions conducted before the age of 10 years were included. This age cut-off was selected to align with the WHO’s definition of adolescence (ages 10–19 years) [34]. Inclusion decisions regarding surgical interventions were guided by the Gardner and Sandberg terminology of ‘urgent’ vs ‘elective’ surgery, with ‘urgent surgeries’ referring to those "performed promptly to avoid life-threatening circumstances or to prevent permanent disability”, while ‘elective surgeries’ include “those that address non-urgent issues” [35].

The following exclusion criteria were developed:

- **Malignancy**. Articles that cited malignancy as their rationale for conducting surgeries were excluded. Some individual congenital variations in sex characteristics are associated with increased risk of germ cell tumours, which is sometimes cited as a rationale for surgical intervention, despite the general malignancy risk associated with congenital variations in sex characteristics being debated [36, 37]. However, for this review, it was thought that the potential for malignancy as perceived by clinicians and presented to parents/guardians, would, in the eyes of concerned parties, change the suggested surgery from being considered ‘elective’ to ‘urgent’. Whether surgical intervention due to the potential risk of malignancy was recommended for the specific congenital variation in sex characteristics in the 2006 *Consensus Statement on Management of Intersex Disorders* [9], which was the last statement to tabulate malignancy risk by individual variation, was recorded, and addressed in the discussion.
- **Urinary incontinence**. Studies citing preserving urinary continence, or studies citing functional urological goals (e.g., removing urethral obstruction), as their rationale were excluded, as these interventions were deemed to be non-elective [35]. Studies whose stated urological goals were intended to bring an individual in line with a perceived normative social behaviour (e.g., surgery to allow a boy to urinate standing up rather than sitting down) were included.

- **Age.** Studies in which it was unclear if all participants were under 10-years-old (i.e., age range was not provided), or studies which reported outcomes for a combination of individuals aged above and below 10-years-old without segregating outcomes, were excluded.
- **No patient outcome data**. Studies that collected no patient outcomes (e.g., studies focusing on surgical equipment use, histological studies) were excluded.
- **Hypospadias, epispadias or cryptorchidism**. Studies addressing hypospadias, epispadias or cryptorchidism that did not present these variations as congenital variations in sex characteristics or did not investigate them as part of an established congenital variation in sex characteristics were excluded due to debate among the medical community over their status as congenital variations in sex characteristics.
- **Primary focus not “sex-normalising” surgical interventions.** Studies that reported patients who had previously received a “sex-normalising” intervention but did not report the outcomes of or rationale for that surgery were excluded.
- **Non-intersex populations**. Studies that reported interventions that could be classed as “sex-normalising” but were administered to patients both with and without (e.g., transgender and gender diverse patients) congenital variations in sex characteristics without segregating outcomes for individuals with congenital variations in sex characteristics, were excluded.
- **Reviews**. Relevant systematic and scoping reviews were excluded but studies included in the reviews were screened against our inclusion criteria as part of a secondary search strategy.

### Data extraction

Data extraction was conducted in *Covidence* [33], using a data-extraction table constructed by LM. LM and LG independently extracted, with CLA extracting a random sample of 25% of studies. In addition to bibliometric data (e.g., author, year of publication), the following data were extracted:

- **Surgical outcomes**. In line with the recommendations for data collection on long-term surgical outcomes for individuals with congenital variations in sex characteristics in the 2018 publication *Caring for Individuals with a Difference of Sex Development [2]*, we recorded if studies reported: complication rates, success rates, cosmetic outcomes, quality of life, sexual functioning, urogenital outcomes, sexuality/gender assignment, re-evaluation, fertility outcomes, social and psychosexual adjustment, mental health, social participation and parental outcomes. The precise and detailed categorisation of long-term outcome reporting used in the 2018 publication was deemed most appropriate for tabulating outcomes, rather than the 2006 *Consensus Statement on Management of Intersex Disorders* [9] or the 2016 *Global Disorders of Sex Development Update since 2006* [4].
- **Rationale**. We recorded whether studies provided a rationale for conducting the surgical intervention, as well as rationale for the timing of the surgery. Rationales were extracted verbatim, alongside any work cited by authors when justifying rationale.
- **Acknowledgement of controversy**. We extracted verbatim text from studies that acknowledged the social, political, cultural, medical, ethical and/or surgical controversy surrounding the intervention they were conducting, as well as any work cited by authors when discussing this controversy.

Quality assessment was conducted independently by LM and CLA using the Joanna Briggs Institute’s instruments for Quality Assessment [38], with consensus reached through discussion. As no syntheses of outcomes were conducted, and rationale and controversy reporting were deemed to be independent of manuscript quality, manuscripts were not excluded on the basis of low quality.

### Data analysis

Descriptive statistics were used to present the number of studies reporting each of the aforementioned outcomes. Qualitative, verbatim data on rationale and controversy was analysed using a narrative synthesis with an inductive approach. LM, LG and AN independently reviewed data to identify codes which addressed commonly occurring themes. LM, LG, and AN met to discuss and agree upon these high-level themes, after which LM independently coded all articles, discussing and refining emerging subthemes with LG and AN.

Throughout the narrative synthesis, the names of congenital variations in sex characteristics used by study authors will be used to avoid unintentional change in diagnosis through misinterpretation by the authorship team. These terms may not always be the accepted term within the intersex or medical communities.

Justifications and rationales for surgical intervention are presented in the narrative synthesis as presented by study teams, regardless of the accuracy of the assumptions stated. The presentation of such rationales does not represent an endorsement of their validity, and the accuracy of stated rationales is explored in the discussion.

## Results

### Search

**Fig 1:**
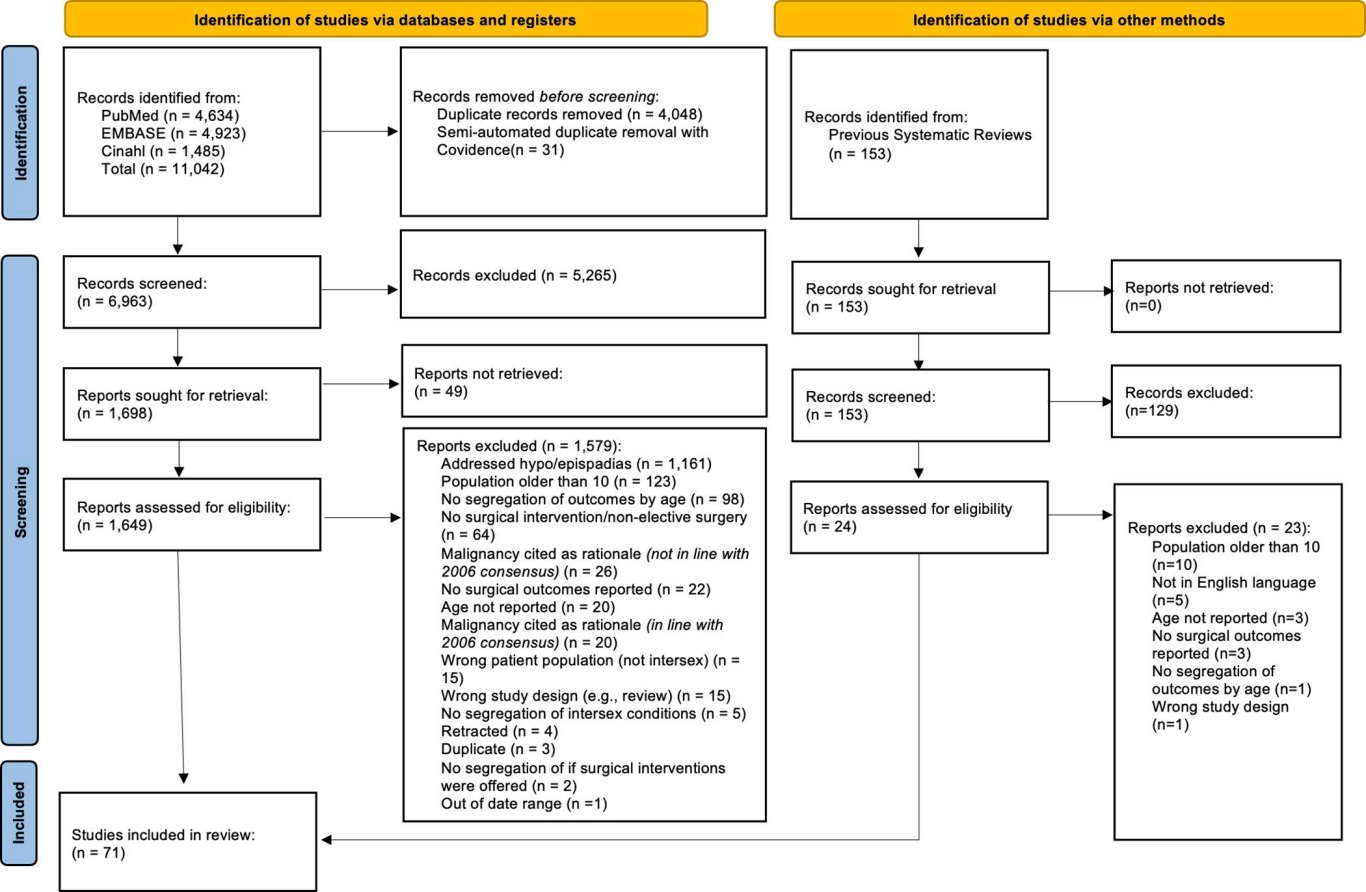
PRISMA flowchart.

Our search yielded 11,042 records, with 6,963 undergoing title/abstract screening after de-duplication. Following full-text screening of 1,649 records, 70 met all inclusion criteria and underwent data extraction. One further study was identified from searching systematic, scoping and literature reviews identified from the search (*Fig 1*). *S2 Text* details the full search results from each database.

Of the 71 studies in the final sample, 73.2% were case studies or case series (n = 52) and 26.8% were cohort studies (n = 19). Countries publishing included studies were India (n = 11; 15.5%), United States of America (n = 7; 9.9%), Brazil (n = 6; 8.5%), Türkiye (n = 5, 7.0%), Japan (n = 4; 5.6%), Algeria (n = 3; 4.2%), United Kingdom of Great Britain and Northern Ireland (UK), Netherlands, Pakistan, Romania, Egypt, Italy (n = 2; 2.8%, respectively), Sweden, Portugal, Republic of Korea, Iran (Islamic Republic of), Canada, Saudi Arabia, Spain, Finland, France, Germany, Indonesia, Argentina, Uganda, Nigeria, China, Poland, North Macedonia, Australia, Iraq, Belgium, Colombia, Kuwait, Syria, Bangladesh, Switzerland and Uzbekistan (n = 1; 1.4%, respectively). Included studies investigated a range of congenital variations in sex characteristics, including 29.6% of studies reporting on congenital adrenal hyperplasia (CAH, n = 21), 15.5% on diphallia and ovotesticular DSD (n = 11, respectively), 8.5% on persistent Mullerian duct syndrome (PMDS, n = 6), 5.6% on mixed gonadal dysgenesis (MGD, n = 4) and 4.2% Mayer-Rokitansky-Küster-Hauser (MRKH) syndrome and partial androgen insensitivity syndrome (PAIS, n = 3, respectively). *Table 1* reports study characteristics. Quality of studies was generally low, with limited description of patient demographics, surgical interventions, and outcomes (*S1 Table*).

**Table 1:** Characteristics of included studies.

Study	Country of first author	Congenital variations in sex characteristics^1^	“Sex-normalising” intervention conducted^2^	Study Design	Sample size (Relevant Sample Size^3^)	Participant Characteristics	Follow-up Time	Rationale for conduct coding	Rationale for conduct (verbatim)	Rationale for timing coding	Rationale for timing (verbatim)
Acimi 2013	Algeria	Congenital adrenal hyperplasia	Vaginoplasty	Case series	27 (18)	Patients with 46 XX, DSD aged 4–28 months. All patients had 21-hydroxylase deficiency, four with simple virilising form and fourteen with additional salt loss. Three patients grade II on Prader scale, five grade III and 10 grade IV and V.	6–48 months	Perceived goal of aligning with assigned sex + Anatomical/Functional/Cosmetic	The objective of feminizing genitoplasty is to allow the child to have a cosmetic aspect of the external genitalia corresponding to gender, a fundamental factor in childhood gender and psychosexual development and separation of the vagina from the urethra with the aim of having a vaginal introitus in the normal perineal position. The neo-vagina must enable the patient to experience in adulthood a normal sexual life, a normal menstrual flow, and even be able to deliver naturally without problems	Perceived goal of aligning with assigned sex + Parental Desire + Anatomical/Functional/Cosmetic	The surgical correction should be performed as early as possible to allow for good development of the patient’s sexual identity […] Several factors support this reasoning: The birth of a child with ambiguous genitalia is a tragedy for the parents, and prolonging this suffering is not justified, the missing part of the vagina is not very important in infants, and early assignment of an appropriate sex of rearing is important for childhood gender and psychosexual development.
Acimi 2018	Algeria	46,XX disorder of sex development, ovotesticular disorder of sex development	Clitoroplasty, vaginoplasty, feminising genitoplasty	Case series	29 (29)	Patients with 46, XX DSD, two with 11-hydroxylase deficiency and twenty-six with 21-hydroxylase deficiency and one ovotesticular DSD between 3–47 months. Four patients had grade II on Prader scale, six grade III and eighteen grade IV and V.	0.5–12 years	Anatomical/Functional/Cosmetic + Perceived goal of aligning with assigned sex	A voluminous glans is often considered ugly due to its disproportionate size compared to the surrounding genitalia, which may be a source of dissatisfaction to the parents and patients […] surgical correction must create the appearance of external genitalia that correspond to the gender.	Anatomical/Functional/Cosmetic	The circumflex arteries of the penis begin to develop, only after the fourth year. This anatomical finding is an argument for performing clitoroplasty at an early age to avoid any risk of intraoperative and postoperative bleeding. […] In patients with 46,XX DSD and some other forms of DSD, the female gender is proposed and surgical correction should be performed as early as possible.
Acimi 2019	Algeria	46, XX disorder of sex development, Ovotesticular disorder of sex development	Vaginoplasty	Case series	22 (22)	Patients with 46, XX DSD, two with 11-hydroxylase deficiency and nineteen with 21-hydroxylase and one ovotesticular DSD between 4–47 months. Eight patients had grade III on Prader scale, fourteen had grade IV and V.	4 months– 10 years	Perceived goal of aligning with assigned sex	In patients with 46, XX DSD and some other types of DSD, the female gender is proposed, and a surgical correction should be performed as early as possible to permit the development of a good gender identity in the patients.	Perceived goal of aligning with assigned sex	A surgical correction should be performed as early as possible to permit the development of a good gender identity in the patients.
Agarwal 2016	India	Mixed gonadal dysgenesis	Laparoscopic gonadectomy with vaginoplasty and clitoral reduction	Case study	1 (1)	7-year-old child with abnormal external genitalia, ovotesticular DSD and 46, XY/45, XO karyotype.	2 years	Perceived goal of aligning with assigned sex	The patient was reared as a girl child because of external genitalia looking like a female. After the operation the same situation was maintained after excising the enlarged clitoris and the child has the potential to become a well-adjusted functional member of the society.	No rationale given	n/a
Agzamkhodjayev 2022	Uzbekistan	Diphallia	Left partial penectomy	Case study	1 (1)	7-year-old child with abnormal genitalia	2 months	No rationale given	n/a	No rationale given	n/a
Akbiyik 2010	Türkiye	Congenital adrenal hyperplasia	Feminising genitoplasty	Case series	41 (41)	Patients aged 1–10 years with 21-hydroxylase deficiency	1 month– 7.5 years	No rationale given	n/a	Perceived goal of aligning with assigned sex + Parental Desire	Surgery in infancy should be aimed principally at creating the appearance of normal external female genitalia, to alleviate parental distress and to avoid the potential psychological squeal of incorrect virilization in girls with CAH […] We now believe that it may be prudent to defer definitive vaginoplasty until puberty. An advantage of deferring vaginoplasty until puberty is the availability of supple, genital skin which provides more robust and amenable flap formation than the genital and introital skin of infants.
Attia 2023	Saudi Arabia	Ovotesticular disorder of sex development	Reconstructive vaginoplasty	Case study	1 (1)	2-month-old baby with abnormal external genitalia and labioscrotal folds	Not reported	Parental Desire	The parents and the team decided to raise the baby as a female. Therefore, the child had reconstructive vaginoplasty.	No rationale given	n/a
Baskin 2020	United States of America	Congenital adrenal hyperplasia, ovotesticular syndrome, mixed gonadal dysgenesis, partial androgen insensitivity syndrome	Feminising genitoplasty	Cohort	57 (50)	Patients with 46, XX DSD due to CAH, partial androgen insensitivity syndrome, mixed gonadal dysgenesis and ovotesticular syndrome	12 months	Anatomical/Functional/Cosmetic	Achieving "typical" female appearance and function in patients with moderate to severe genital atypia	No rationale given	n/a
Bernabé 2018	United States of America	Gonadal dysgenesis, partial androgen insensitivity syndrome, severe hypospadias and microphallus, congenital adrenal hyperplasia, chromosome mosaicism	Masculinising and feminising genitoplasty	Cohort	27 (27)	Ten 46,XY patients with gonadal dysgenesis, partial androgen insensitivity syndrome, testosterone biosynthetic defect, severe hypospadias and microphallus. Sixteen 46, XY patients with CAH. One child with sex chromosome mosaicism (45,X/46,XY)	12 months	No rationale given	n/a	No rationale given	n/a
Birraux 2015	Switzerland	Congenital adrenal hyperplasia	Feminising genitoplasty	Case study	1 (1)	3-year-old child with genital ambiguity, severe virilisation, Prader V stage and 46, XX karyotype	2 months	No rationale given	n/a	No rationale given	n/a
Boia 2014	Romania	Antley-Bixler Syndrome	Combined perineal and transabdominal surgical intervention for ambiguous genitalia	Case study	1 (1)	3-year-old child with facial dimorphism, forearm and elbow malformations and ambiguous genitalia	6 months	No rationale given	n/a	No rationale given	n/a
Bose 2022	India	5-alpha reductase deficiency	Single stage urethroplasty, bilateral orchidopexy, correction of penoscrotal transposition, urethrocutaneous fistula closure, chordee correction, excision of UGS remanent, partial urethral tubularisation, excision of urogenital sinus remnant, release of right obstructed inguinal hernia	Case series	12 (10)	5-alpha reductase deficiency cases managed at a paediatric gender clinic ranging from 3 days-14 years old	4–10 years	No rationale given	n/a	Perceived goal of aligning with assigned sex + Anatomical/Functional/Cosmetic + Parental Desire	The younger the child at gender assignment, the better is the adjustment of the child/family to provide support for the management decision […] Late diagnosis leads to female sex of rearing, feminizing surgery and gonadectomy in childhood or incongruous pubertal virilization in those with retained gonads. Subsequent pharmacological induction of puberty and infertility culminates in poor outcome in adolescence and adulthood […] in the sociocultural context we practice in, the parents/caretakers are anxious to complete the procedures in early childhood before schooling and peer interactions.
Braga 2011	Canada	Persistent cloaca	Posterior sagittal anorectoplasty and total urogenital sinus mobilisation	Case study	1 (1)	34-week gestation new-born with XX karyotype presenting with an enlarged clitoris, bilateral non-palpable gonads, and a single perineal orifice at 10 months	6 months	Anatomical/Functional/Cosmetic	Allowed a more natural looking, and possibly functioning, vaginal introitus, improving the final cosmetic result […] Creating more normal-looking female external genitalia	No rationale given	n/a
Chowdhury 2018	Bangladesh	Ambiguous genitalia	Orchiopexy, separation and closure of vaginal orifice, chordee correction, urethroplasty, scrotal reconstruction	Case study	1 (1)	8-months-old child presenting with enlarged clitoris, bifid scrotum and separate presence of urethral and vaginal orifices	Lost to follow-up	Perceived goal of aligning with assigned sex	To convert her into male sex	No rationale given	n/a
CorrêaLeite 2014	Brazil	Diphallia	Penectomy	Case study	1 (1)	2-year-old boy with 46, XY karyotype presenting with two separate penises	2 years	No rationale given	n/a	No rationale given	n/a
Correya 2021	India	Gonadal dysgenesis	Hypospadias correction	Case series	2 (2)	6-month-old presenting with penoscrotal hypospadias, micropenis and right non-palpable testis	No follow-up	No rationale given	n/a	No rationale given	n/a
Dangle 2017	United States of America	Congenital adrenal hyperplasia	Genitourinary reconstructive surgery	Cohort	26 (26)	Patients with CAH aged 5–87 months old, with all but two diagnosed with classical CAH, with the other two diagnosed with non-classical	4.5–142 months	Anatomical/Functional/Cosmetic	Favourable cosmetic and functional outcomes	Anatomical/Functional/Cosmetic + Perceived goal of aligning with assigned sex	Proponents of early repair support it on the basis of maternal estrogen effect, minimizing parenteral stress, better compliance, and no recollection of surgery later in adulthood. Others propose delayed intervention during adolescent age or beyond due to additional surgical interventions required following childhood reconstruction
Dehneh 2022	Syrian Arab Republic	Congenital adrenal hyperplasia	Reconstructive surgery	Case series	5 (4)	46, XX Infant with male-appearing external genitalia and untraceable testes, 46, XX 5-year-old with ambiguous genitalia with adrenal hypertrophy and vagina and 46, XX infant with ambiguous genitalia	Not reported	Perceived goal of aligning with assigned sex + Anatomical/Functional/Cosmetic	Reconstructive surgery […] could offer psychological relief, by resolving the sexual ambiguity of the genitalia, and may facilitate sexual intercourse, although it may enhance the feeling of being different	Belief of Best Practice	Early surgical treatment, rather than delayed or staged approaches for 46, XX CAH patients with specific degrees of genital virilization, has been included in guidelines for the Development of Comprehensive Care Centers for Congenital Adrenal Hyperplasia.
Deshpande 2020	India	Diphallia	Amputation of the left phallus	Case report	1 (1)	2-year-old with two well-formed penises	10 months	No rationale given	n/a	No rationale given	n/a
Elsawy 2012	Kuwait	Diphallia	Surgical reconstruction	Case study	1 (1)	37-day-old with duplicated penis	1 year	No rationale given	n/a	No rationale given	n/a
Elsayed 2020	Egypt	Congenital adrenal hyperplasia	Urogenital sinus mobilisation and nerve-sparing clitoroplasty	Cohort	61 (35)	61 children who underwent feminising genitoplasty, 35 of whom had this conducted before 2 years of age (aged between 3 days-10 months), and 26 after.	1–12 years	Anatomical/Functional/Cosmetic	Correct the external genitalia and separate the genital from urinary tract with correction of the external genitalia achieving a desirable cosmetic and functional outcome	No rationale given	n/a
Erginel 2023	Türkiye	Congenital adrenal hyperplasia	Clitoroplasty and vaginoplasty	Cohort	14 (14)	14 patients aged 10–96 months who underwent feminising genitoplasty, 7 of whom were under the age of 2	3–18 years	Anatomical/Functional/Cosmetic	To create a female-like external genitalia, including an introitus that will allow intercourse, ensure menstrual flow […] to promote the female gender and to avoid psychological stress on the parents and girls	Perceived goal of aligning with assigned sex	Before two years of age, children are psychosocially neutral; it is important to perform the operations before this age because the child is not likely to remember the surgery. The reconstructive process would have been completed before sexual identity was established […] performed infant genital surgeries promote the female gender and to avoid psychological stress on the parents and girls
Fares 2019	Egypt	Congenital adrenal hyperplasia	Surgical management of high urogenital sinus	Cohort	7 (7)	Patients aged between 12 months-5 years with a preconfluence urethra of < 15mm	12–18 months	Anatomical/Functional/Cosmetic	Aiming to produce a feminine appearance, preserving the delicate genital nerve supply	Perceived goal of aligning with assigned sex	Patients classified according to Prader’s classification (from III to V degrees), would arguably need a form of clitoroplasty. One of the established strategies is a single-stage early feminizing surgery, aiming to produce a feminine appearance, preserving the delicate genital nerve supply […] aiming to confer an early physical appearance consistent with the female gender of rearing, and to cause less psychological stigmatization than with delayed surgery.
Fernandez 2021	Colombia	Congenital adrenal hyperplasia	Complete corporeal preservation clitoroplasty, vaginoplasty	Cohort	4 (4)	Patients with mean age of 18.5 months and a molecular confirmed diagnosis of CAH	1 year	Anatomical/Functional/Cosmetic + Belief of Best Practice	Restore female aspect of genitalia while preserving dorsal neurovascular bundle but not at the expense of not preserving erectile tissue […] In the last consensus statement on the management of DSD, it was suggested to perform early surgery and UGS repair in girls with severe virilization	Perceived goal of aligning with assigned sex + Parental Desire + Anatomical/Functional/Cosmetic + Belief of Best Practice	Most families opt for early surgery, considering the positive implications for children’s psychosocial development, relieving parents distress and restoring “normal” external genital configuration […] The 4th World Congress of the International Society of Hypospadias and Disorders of Sex Development Surgery and the American Academy of Pediatrics, suggested performing the surgery before 2 years of age.
Ferong 2020	Belgium	45,X/46,XY disorder of sex development	Resection of gonadal structure	Case series	6 (1)	New-born with diagnosis of 45,X/46,XY DSD referred for management of penoscrotal hypospadias and non-palpable testis	6 months	No rationale given	n/a	No rationale given	n/a
Fukui 2012	Japan	Ovotesticular disorder of sex development	Herniorrhaphy and removal of right gonad, uterus and fallopian tubes with one-stage hypospadias repair	Case study	1 (1)	1-year-old child presenting with hypospadias, right undescended testes and asymmetric external genitalia	Not reported	No rationale given	n/a	No rationale given	n/a
Garge 2014	India	Herlyn-Weber-Wunderlich Syndrome	Septoplasty to create a single vaginal orifice	Case study	1 (1)	10-year-old child presenting with well-defined mass in suprapubic region with vulvar mass and a normal vaginal and urethral orifice	2 months	No rationale given	n/a	No rationale given	n/a
Gozar 2014	Romania	Congenital adrenal hyperplasia	Clitoroplasty, reconstruction of labia minora, creation of neovulva and vaginoplasty	Case study	1 (1)	3-year-old child presenting with history of classic CAH, with external genital pigmentation, clitoromegaly and labioscrotal fusion	4 years	Anatomical/Functional/Cosmetic + Perceived goal of aligning with assigned sex	To help normal psychosexual development and creation of a functional vagina to allow menstruation and sexual activity	Belief of Best Practice	The Endocrine Society suggests that in patients with a low vaginal confluence, complete repair, including vaginoplasty, perineal reconstruction, and clitoroplasty (if necessary), can be done at an early age
Gupta 2018	India	Persistent Müllerian duct syndrome	Left herniotomy with bilateral trans-septal orchidopexy	Case study	1 (1)	1-year-old with swelling in left inguinal region and absent right testis since birth	2 days	No rationale given	n/a	No rationale given	n/a
Jesus 2018	Brazil	Genital ambiguity	Feminising genitoplasty with total urogenital sinus mobilisation	Cohort	8 (7)	Six patients with CAH and two with mixed gonadal dysgenesis, with a mean age of surgery of 51 months	3–56 months	Belief of Best Practice + Anatomical/Functional/Cosmetic	According to the Chicago Consensus 2006, feminizing genitoplasty, when indicated, should be performed in the most virilized cases (Prader III to V) and should be performed in specialized centers, with the focus being on future sexual function, not just cosmetic appearance […] this technique, when indicated, is feasible and effective in achieving the objectives of this type of surgery; i.e. adequate separation between the vagina and urethra to achieve high urethrovaginal confluence, a well-positioned clitoris and a satisfactory external appearance of the vagina.	No rationale given	n/a
Joshi 2007	United Kingdom of Great Britain and Northern Ireland	Mixed gonadal dysgenesis	Laparoscopic excision of Mullerian structures	Case study	1 (1)	2-day old neonate presenting with proximal hypospadias and a non-palpable right gonad	2 days	Perceived goal of aligning with assigned sex	As gender assignment had already been agreed, we undertook an early excision of the Mullerian structures	Anatomical/Functional/Cosmetic	Considering the size of the hydrometrocolpos, the potential risk of infection, and the pressure effects over adjacent structures, we undertook an early surgical intervention […] The failure to recognize and promptly manage a hydrocolpos can lead to pyocolpos, vaginal perforation, persistent bilateral hydronephrosis, megaureters, recurrent urinary tract infections, persistent acidosis, and a failure to thrive. The size of the hydrometrocolpos in this patient prompted us to decide on an early surgical intervention
Kamble 2015	India	Transverse testicular ectopia with persistent Müllerian duct syndrome	Laparoscopic correction with division and removal of uterus	Case study	1 (1)	4-month-old with non-palpable right testis and left sided inguinal hernia	6 months	No rationale given	n/a	No rationale given	n/a
Keir 2009	United Kingdom of Great Britain and Northern Ireland	Congenital adrenal hyperplasia	Removal of Mullerian structures	Case study	1 (1)	5-year-old with micropenis, penile hypospadias and impalpable testes	Not reported	Parental Desire	The parents, particularly the father, expressed a strong desire for removal of the Mullerian organs. Given the recurrent abdominal pain and the presence of haematocolpos, there may have been a clinical basis for surgery, but initially the clinical team discouraged it, explaining that at this young age the procedure is permanent and cannot be reversed. However, as the family was returning to their country of origin where this procedure may have been performed by less experienced surgeons, the clinical team proceeded to surgery to remove the patient’s Mullerian organs.	Access	Ultimately, because the patient and family were only temporary visitors to the UK, in the interests of the child we decided to perform the surgery in our expert unit rather than risk having it performed at a non-specialist facility in the country of origin
Kendrick 2021	Australia	Diphallia	Excision of the right phallus	Case study	1 (1)	Neonate presenting with penile duplication	Not reported	No rationale given	n/a	No rationale given	n/a
Kirli 2013	Türkiye	Congenital adrenal hyperplasia	Hysterectomy with bilateral salphingo-oophorectomy and vaginectomy, chordee release, urethra repair, surrenalectomy, mastectomy, fistula repair	Cohort	11 (11)	Patients ranging from 5 days-10 years, presenting with nonpalpable gonads, hyperpigmentation, jaundice and electrolyte imbalance, all with non-palpable gonads	Not reported	Perceived goal of aligning with assigned sex + Anatomical/Functional/Cosmetic	Because genital appearance is one of the main factors of normal sexual development, successful genital surgery is the important step of treatment […] In this situation, discordance between genetic gender and phenotypic sexual characteristics faces the gender assignment team including paediatric surgeon to a conflict. Unfortunately, paediatric surgeon is obligated to plan corrective surgery of masculinising genitoplasty […] Aim of the treatment is to provide normal physical and psychosocial development with protection of fertility of the patient whenever possible […] In a case with delayed diagnosis of 46XX virilising CAH, implementation of male reconstructive surgery can provide satisfactory male gender identity and heterosexual orientation if phenotype and chosen identity is male.	Perceived goal of aligning with assigned sex + Parental Desire + Anatomical/Functional/Cosmetic	Early diagnosis and management of intersex disorder is essential to provide normal metabolic, physical and psychosocial development […] In our opinion, surgical procedures should be performed as soon as the gender is assigned for the comfort of child and family.
Kocova 2019	North Macedonia	5-alpha reductase deficiency	Orchidectomy and clitoroplasty	Case study	2 (1)	20-month-year old with rugged labia resembling scrota, phallus and a perineal opening	20 years	No rationale given	n/a	Perceived goal of aligning with assigned sex	Timing of putative sex reversal is important, for example, 5-ARD deficiency has been confirmed in female athletes excelling in athletics, thus complicating their sports life […] The recent guidelines should be followed in 46,XY under virilized babies, and leave the possibility for sex reversal for later in life, if needed.
Kudela 2020	Poland	Congenital adrenal hyperplasia	Feminising genitoplasty	Cohort	31 (31)	Group with mean age of 19 months consisting of 29 with 21-hydroxylase deficiency and 2 with 11-beta-hydroxylase deficiency, 7 having Prader grade III external virilisation, 21 with grade IV and 3 with grade V	12 months-15 years	Perceived goal of aligning with assigned sex + Parental Desire + Anatomical/Functional/Cosmetic	Female gender identity can be expected in a person with 46,XX karyotype and CAH, therefore early feminizing genitoplasty in these cases seems to be justified. Surgical correction of even very severe virilization in female patients with CAH can restore female-pattern appearance of the genitalia […] All parents demanded early genital reconstructions despite the information from the multidisciplinary team about the option of postponing the reconstructive operation until the age of consent […] The purpose of the operation is to restore proper functional and female-looking anatomy of the genitalia.	Parental Desire + Belief of Best Practice	Female gender identity can be expected in a person with 46,XX karyotype and CAH, therefore early feminizing genitoplasty in these cases seems to be justified […] According to the current Endocrine Society guidelines in minimally virilized girls, observation or delayed surgery are preferred, however in severe virilized CAH females, early reconstruction is recommended […] Patients with CAH and 46,XX karyotype usually have no gender identity problems. The rare exceptions are lately diagnosed severely virilized cases who were assigned at birth and raised as male. Therefore, the majority of parents of 46,XX CAH patients desire early corrective surgery […] Similar to hypospadias, which is another much more common defect of the genitalia, it is probably better to perform the genital reconstruction between 6 to 18 months of age. This age is believed to be the best for corrective surgery based on psychological aspects […] Although there is no clear evidence in the literature showing that early surgery of 46,XX CAH is superior to late surgery, we believe that atypical genitalia including large clitorises may cause much psychological harm to patients and their families.
Kumar 2015	India	True hermaphroditism	Total abdominal hysterectomy, bilateral salphingo-oophorectomy and colpectomy	Case study	1 (1)	3-year-old child admitted with ambiguous genitalia, with swellings in inguinal region, non-visible testis and scrotum and presence of enlarged clitoris	Not reported	Perceived goal of aligning with assigned sex	Child was made after extirpation of female genitals […] If the patient is to be raised as female, all testicular and wolffian tissues should be removed	Anatomical/Functional/Cosmetic	If a male gender is assigned, as has been most common historically, all ovarian and Mullerian tissue should be removed.
Kundal 2013	India	Diphallia	Penile amputation and phalloplasty	Case study	1 (1)	3-year-old presenting with two separate phalluses	Not reported	No rationale given	n/a	No rationale given	n/a
Levy 2023	United States of America	5-alpha reductase deficiency	Staged hypospadias repair	Case study	1 (1)	Infant presenting with 2-cm genital tubercle with a single perineal opening and bifid labioscrotal folds	Not reported	No rationale given	n/a	No rationale given	n/a
Liu 2010	China	Congenital adrenal hyperplasia	Clitoroplasty, reconstruction of labia minora	Case study	1 (1)	4-year-old child presenting with enlarged, hypertrophied clitoris	Not reported	Anatomical/Functional/Cosmetic + Perceived goal of aligning with assigned sex + Parental Desire	To restore the female’s external genital appearance and function […] Many reports emphasize the importance of reconstructing the clitoris to restore its physical appearance and its sensitivity to sexual stimuli […] The larger erectile clitoris can embarrass the parents and bring pain to the child, passively influencing development of the sexual psyche. Meanwhile, the absence of a vagina will not affect a small child. Sometimes, restoration of the vulva’s appearance is more important to the female pseudohermaphrodite girl than vaginal reconstruction.	Perceived goal of aligning with assigned sex + Anatomical/Functional/Cosmetic	Considering the deficiency and hypogenesis of the local tissue, surgical treatment was immediately considered due to the child’s mental development […] Several factors influence the timing of elective gender remodelling, including age-related aesthetics and surgical risks and benefits in relation to the psychosexual impact of the procedure during the various stages of development. Performing a genitoplasty before the age of 30 months seems to be important because awareness of sexual identity begins at that time […] The repair of genital malformation as early as possible will help patients to achieve a psychologically healthy body image
Macedo 2009	Brazil	Transverse testicular ectopia and persistent Müllerian duct syndrome	Excision of Mullerian remnant	Case study	1 (1)	1-year-od child presenting with bilateral cryptorchidism with a uterus	Not reported	No rationale given	n/a	No rationale given	n/a
Macedo 2015	Brazil	Congenital adrenal hyperplasia	Total urogenital mobilisation	Case study	1 (1)	9-month-old child presenting with Prader III virilisation of external genitalia and 46, XX karyotype	Not reported	No rationale given	n/a	No rationale given	n/a
Macedo 2022	Brazil	Diphallia	Removal of left penile	Case study	1 (1)	2-year-old child with penile duplication	Not reported	Anatomical/Functional/Cosmetic	The goal of attaining satisfactory functional and cosmetic results.	No rationale given	n/a
Matsui 2011	Japan	Ovotesticular disorder of sex development	Clitoroplasty, vaginoplasty, urethroplasty, scrotoplasty and uterocolpectomy	Cohort	8 (8)	Children presenting at mean age of 2.4 months, with ambiguous genitalia, isolated clitoromegaly, perineal hypospadias and cryptorchidism	3–16 years	Perceived goal of aligning with assigned sex	Surgery is generally necessary after gender assignment. This includes removal of gonads and internal ducts inappropriate to the sex of rearing, and genitoplasty to construct the appropriate external appearance.	Parental Desire + Perceived goal of aligning with assigned sex	Despite informing families of all treatment options available, they have often desired early gonadal surgery and genitoplasty. We believe early operations improve the attachment between a child and parents and benefit the development of gender identity in childhood.
Matsumoto 2012	Japan	Ovotesticular disorder of sex development	Resection of ovarian segment	Case study	1 (1)	7-year-old child with ascent of scrotal contents, 46, XX karyotype and history of Mullerian remnant resection	7 years	No rationale given	n/a	No rationale given	n/a
Matsumoto 2016	Japan	Diphallia	Surgical correction of penis and urethra	Case study	1 (1)	9-month-old child presenting with two separate phalluses bifid scrotum and hypospadic urethral meatus	5 years	Anatomical/Functional/Cosmetic	To achieve good functional and cosmetic outcomes	No rationale given	n/a
Mirshemirani 2010	Iran (Islamic Republic of)	Diphallia	Reimplantation and resection of the left phallus	Case study	1 (1)	2-day-old chid presenting with with duplicated penis and proximal hypospadias on left penile	4 years	No rationale given	n/a	No rationale given	n/a
Nasir 2019	Nigeria	Disorders of sexual development	Urethroplasty, penis straightening, scrotoplasty, orchidopexy, feminising genitoplasty, gonadectomy	Cohort	15 (14)	Fifteen children presenting with DSDs with a median age of 20 months. Ten presenting with ambiguous genitalia and five presenting with hypospadias	2–26 months	Perceived goal of aligning with assigned sex	Accurately and appropriately assign sex	Perceived goal of aligning with assigned sex	A delay in making gender assignment or reassignment of the wrong gender is often fraught with emotional trauma and psychosocial issue […] it is generally agreed that the diagnosis of DSD should be promptly established after delivery and preferably before discharge so that an early sex of rearing can be assigned to an affected child and treatment can be planned
Nokoff 2017	United States of America	Disorders of sex development	Feminising and masculinising genitoplasty	Cohort	37 (35)	Children presenting with DSDs ranging from 5.4–29.7 months old. Twenty children had a 46, XX karyotype, fifteen had 46, XY and two had chromosome mosaicism	6 months	Belief of Best Practice	It is recommended that genital surgery for a child raised as a female only be considered in cases of severe virilization (Prader 3–5) and that surgery of the clitoris not be performed for reasons of cosmetic appearance alone	Belief of Best Practice + Anatomical/Functional/Cosmetic	It is recommended that clitoral and perineal reconstruction be considered in infancy and those with a low vaginal confluence undergo vaginoplasty at an early age; the appropriate timing is less certain for those with a higher vaginal confluence […] Hypospadias repair is more successful if performed in pediatric rather than adult patients
NoumanAli 2022	Pakistan	Diphallia	Distal penis resection	Case study	1 (1)	10-year-old presenting with double penis since childhood	Not reported	Anatomical/Functional/Cosmetic	To achieve normal contour of the genitalia	No rationale given	n/a
Oyania 2023	Uganda	Mayer-Rokitansky-Küster-Hauser Syndrome	Vaginal reconstruction	Case study	1 (1)	3-year-old presenting with sigmoid colostomy and unrecognised MRKH syndrome	Not reported	Anatomical/Functional/Cosmetic	Creating a new cavity and replacing the vagina with a mucous membrane lined canal such as a segment of bowel	No rationale given	n/a
Ozsu 2013	Turkey	Ovotesticular disorder of sexual development	Left gonadectomy and hypospadias repair	Case study	1 (1)	5-month old presenting with hypospadias, cryptorchidism and micropenis, with ovarian tissue, fallopian tubes and uterine remnants in left inguinal canal	11 years	No rationale given	n/a	No rationale given	n/a
Ozturk 2007	Türkiye	Persistent Müllerian Duct Syndrome with transverse testicular ectopia	Hysterectomy with resection of underdeveloped fallopian tubes	Case series	2 (2)	8-month-old child presenting with right incarcerated inguinal hernia and an empty left hemiscrotum	Not reported	No rationale given	n/a	No rationale given	n/a
Parelkar 2009	India	Persistent Müllerian duct syndrome	Laparoscopic orchidopexy	Case study	1 (1)	10-month old infant presenting with left inguinal hernia and bilateral nonpalpable gonads	6 months	Anatomical/Functional/Cosmetic	Placement of well-vascularized testes in the scrotum	No rationale given	n/a
Park 2011	Republic of Korea	Genital ambiguity with high vaginal confluence.	Feminising genitoplasty, total urogenital mobilisation	Cohort	10 (7)	Patients with median age at time of surgery of 21 months, seven with CAH, one with mixed gonadal dysgenesis, one with partial androgen insensitivity and one with 5-alpha reductase deficiency syndrome	3–12 years	Anatomical/Functional/Cosmetic	Provide a normal cosmetic appearance without sacrificing sensation or vascularity of the glans, due to the importance of the clitoris in female sexual response and in achieving orgasm.	No rationale given	n/a
Paula 2015	Brazil	Ovotesticular disorder of sex development	Left gonadectomy, removal of Mullerian structures and urethroplasty	Case study	1 (1)	2-month-old child with Prader stage III virilisation of external genitalia	5 years	No rationale given	n/a	No rationale given	n/a
Podesta 2008	Argentina	Congenital adrenal hyperplasia, partial androgen insensitivity, mixed gonadal dysgenesis	Feminising genital reconstruction	Cohort	12 (12)	Patients ranging from 0.4–5.3 years old presenting with high entrance of vagina into the urethra	3–12 years	Anatomical/Functional/Cosmetic	Give the urinary tract and genital apparatus as normal an anatomical state as possible, while also providing physiologic functions […] producing an aesthetically pleasing feminine genital appearance	No rationale given	n/a
Rahayatri 2021	Indonesia	Mayer-Rokitansky-Küster-Hauser	Vaginal reconstruction	Case study	1 (1)	15-month-old presenting with transverse colostomy and clitoromegaly	2 weeks	Perceived goal of aligning with assigned sex	Reduce the impact that this condition may have on physiological processes in the future.	No rationale given	n/a
Rehman 2020	Pakistan	Congenital adrenal hyperplasia	Feminising genitoplasty	Cohort	32 (32)	Patients aged 6–18 months, 3 with Prader Scale grade I external virilisation, 6 with grade II, 13 with grade III and 10 with grade IV	3 weeks	Anatomical/Functional/Cosmetic + Perceived goal of aligning with assigned sex	As the children retain the potential for normal sexual activity and fertility in classical CAH, the treatment is directed to attain anatomical and psychological female gender and for this, children undergo feminizing genitoplasty after detailed diagnostic assessment and counselling	No rationale given	n/a
Roll 2006	Germany	Congenital adrenal hyperplasia	Complete one-stage genital reconstruction, clitoroplasty,	Cohort	19 (19)	Patients aged 1-7-years-old, seventeen with salt-wasting CAH and one with simple virilisation	6 months-32 years	Anatomical/Functional/Cosmetic	A normal looking sensate clitoris, an adequately sized and appropriately situated vagina and a good functional outcome are the main goals of feminising genital reconstructive surgery.	Perceived goal of aligning with assigned sex + Parental Desire	Will be performed as early as possible to prevent psychological disturbances in the children and the parents and to avoid delay of the vaginoplasty procedure
Samadi 2021	United States of America	Diphallia	Phalloplasty	Case study	1 (1)	10-month-old with duplicated penis	3 months	No rationale given	n/a	No rationale given	n/a
Savanelli 2008	Italy	Congenital adrenal hyperplasia	Feminising genitoplasty	Cohort	14 (14)	Patients aged between 6 months and 4 years, five of whom had a vaginal orifice next to the external urethral sphincter and nine presenting with a more distal form	3 months-8 years	Belief of Best Practice + Anatomical/Functional/Cosmetic + Parental Desire + Perceived goal of aligning with assigned sex	Recent consensus statement on the management of intersex disorders suggests that cosmetic surgery in girls with severe virilization (Prader III to V) should be performed in the first year of life, when appropriate, in conjunction with common UGS repair […] many believe that early surgery is appropriate in these children for a better and malleable tissue. We privilege a full feminization in the first years of life in distal and high forms […] The excellent anatomical appearance of the vulva created at an early stage facilitates the parents’ acceptance and helps create a more normal parents-daughter attachment and family relationships.	Perceived goal of aligning with assigned sex + Anatomical/Functional/Cosmetic	There are also controversies regarding the need for clitoral reduction. However, leaving a grossly enlarged clitoris untouched during childhood underscores the psychological impact that this situation can cause to the untreated child […] many believe that early surgery is appropriate in these children for a better and malleable tissue
Scarpa 2019	Italy	Ovotesticular differences of sex development	Orchiopexy and hypospadias repair, removal of testicular tissue	Case series	3 (3)	Three patients aged 12–17 months presenting with genital ambiguity	3 months	Perceived goal of aligning with assigned sex	Surgery is necessary after gender assignment and includes removal of gonads and internal ducts inappropriate to the sex of rearing and genitoplasty to construct the appropriate external appearance […] The fertility potential must be respected and a satisfactory result must be obtained […] we removed the female gonad because of its macroscopic aspect of streak gonad with a potential tendency to degeneration. Although the fertility potential in a male OT-DSD is doubtful, the future presence of estradiol in developing ovarian follicles could inhibit spermatogonia development in contralateral seminiferous tubules. There is no evidence that prophylactic removal of asymptomatic Müllerian remnants is required. If possible, we suggest leaving them in situ in male patients. In cases 2 and 3 we maintained the ovarian part of the ovotestis for preserving a potential fertility even if in case 3 the uterus was not found. The families of the last two cases were strongly oriented to a female sex of rearing.	Parental Desire	Families often ask for an early surgical solution to ensure child well-being within the family, the school and the society.
Sekhon 2017	India	Persistent Müllerian Duct Syndrome	Excision of uterus, fallopian tubes and portion of vagina	Case study	1 (1)	Two-month-old infant presenting with left irreducible inguinal hernia with right non-palpable undescended testis	Not reported	Anatomical/Functional/Cosmetic	Reducing the impact of various medical, social and psychological problems associated with this condition […] If PMDS is discovered as an unexpected finding while operating, biopsy of the gonadal tissue and Mullerian structures is warranted […] Parental counselling about the complex pathophysiology should be done, keeping in mind the psychosocial implications. Once the diagnosis of PMDS is confirmed, the surgical management consists of excision of Mullerian remnants with orchidopexy.	Anatomical/Functional/Cosmetic	Performing definitive surgery at an early age, thereby reducing the impact of various medical, social and psychological problems associated with this condition
Tran 2011	United States of America	Ovotesticular disorder of sex development	Total abdominal hysterectomy, vaginectomy, bilateral gonadectomy, first-stage hypospadias repair	Case study	1 (1)	1-day-old infant presenting with fused bifid labioscrotal folds, impalpable gonads, small phallus with severe chordee and penoscrotal hypospadias	19 months	Perceived goal of aligning with assigned sex	Removal of gonads and internal genitalia that oppose the assigned sex	No rationale given	n/a
Tuna 2019	Portugal	Herlyn-Werner-Wunderlich Syndrome	Vaginal septotomy	Case study	1 (1)	4-day-old infant presenting with absent right kidney, pelvic cystic lesion and a bulging in the location of the vaginal introitus	4 days	Anatomical/Functional/Cosmetic	Obstructive reproductive tract anomalies, such as HWWS, comprise a higher risk of hematosalpinx, endometriosis, and pelvic inflammatory disease, potentially threatening the fertility of these patients […] The preferred treatment consists in the excision of the obstructing vaginal septum.	Anatomical/Functional/Cosmetic	Early and accurate diagnosis and treatment are of the utmost importance to avoid complications and maintain the reproductive potential of the patients […] Our clinical case shows that prenatal suspicion and careful physical examination at birth allows early diagnosis and management of HWWS, which relates to better outcomes and avoidance of potential lifelong complications.
VanDerZwan 2013	Netherlands	Disorders of sex development	Hypospadias correction, gonadectomy, orchiopexy,	Cohort	14 (14)	Participants aged between 14-32-years-old who underwent surgical intervention between ages of 1-6-years-old with partial androgen insensitivity syndrome, mixed gonadal dysgenesis, ovotesticular DSD and undefined 46, XY DSD	Not reported	Anatomical/Functional/Cosmetic	The aim of masculinizing surgery in patients with disorders of sex development is to improve cosmesis and function of the external genitalia, to enable sexual intercourse and to avoid stigmatization	No rationale given	n/a
Vivier 2011	France	Herlyn-Werner-Wunderlich syndrome	Trans-hymenal resection of vaginal septum	Case study	1 (1)	1-month-old with dilated left pelvic kidney, uterus didelphys and communication between the left hemi-uterus and a retrovesical fluid-filled pelvic structure	1 month	Anatomical/Functional/Cosmetic	To prevent complications such as hemihaematocolpos and secondary endometriosis.	No rationale given	n/a
Wester 2012	Spain, Finland, Sweden	Mayer-Rokitansky-Küster-Hauser (MRKH) syndrome	Sigmoid colovaginoplasty, vaginal pull-through	Case series	7 (6)	Patients aged between 13 months-17.5 years presenting with MRKH syndrome	1 month-1 year	No rationale given	n/a	Anatomical/Functional/Cosmetic	In patients with vaginal agenesis and anorectal malformation, the vagina is normally created at the time of anorectal reconstruction. The rationale for this is that the perineum and the tissue plane between the urinary tract and rectum is scarred after the anorectal reconstruction, making a secondary procedure more difficult.
Wolffenbuttel 2019	Netherlands	Perineal hypospadias in disorders of sex development	Perineal hypospadias repair	Case series	4 (4)	Patients aged between 6 monhts-5 years presenting with perineal hypospadias associated with a range of DSDs	6 months-5 years	Anatomical/Functional/Cosmetic	The main rationale for adopting this conservative approach however is to minimize genital tissue removal in children with a not yet definite gender identity, which will certainly facilitate unforeseen future gender reassignment surgery	No rationale given	n/a

Notes

1. Terminology used for congenital variations in sex characteristics matches those reported by authors and does not necessarily reflect the current or correct terminology used in medical practice or by the intersex community.

2. Terminology used for surgical interventions matches those reported by authors and does not necessarily reflect the current or correct terminology used in medical practice or by the intersex community.

3. Relevant sample size is the number of patients within a cohort or case series that meet all inclusion criteria.

### Outcomes measured in the literature

**Fig 2:**
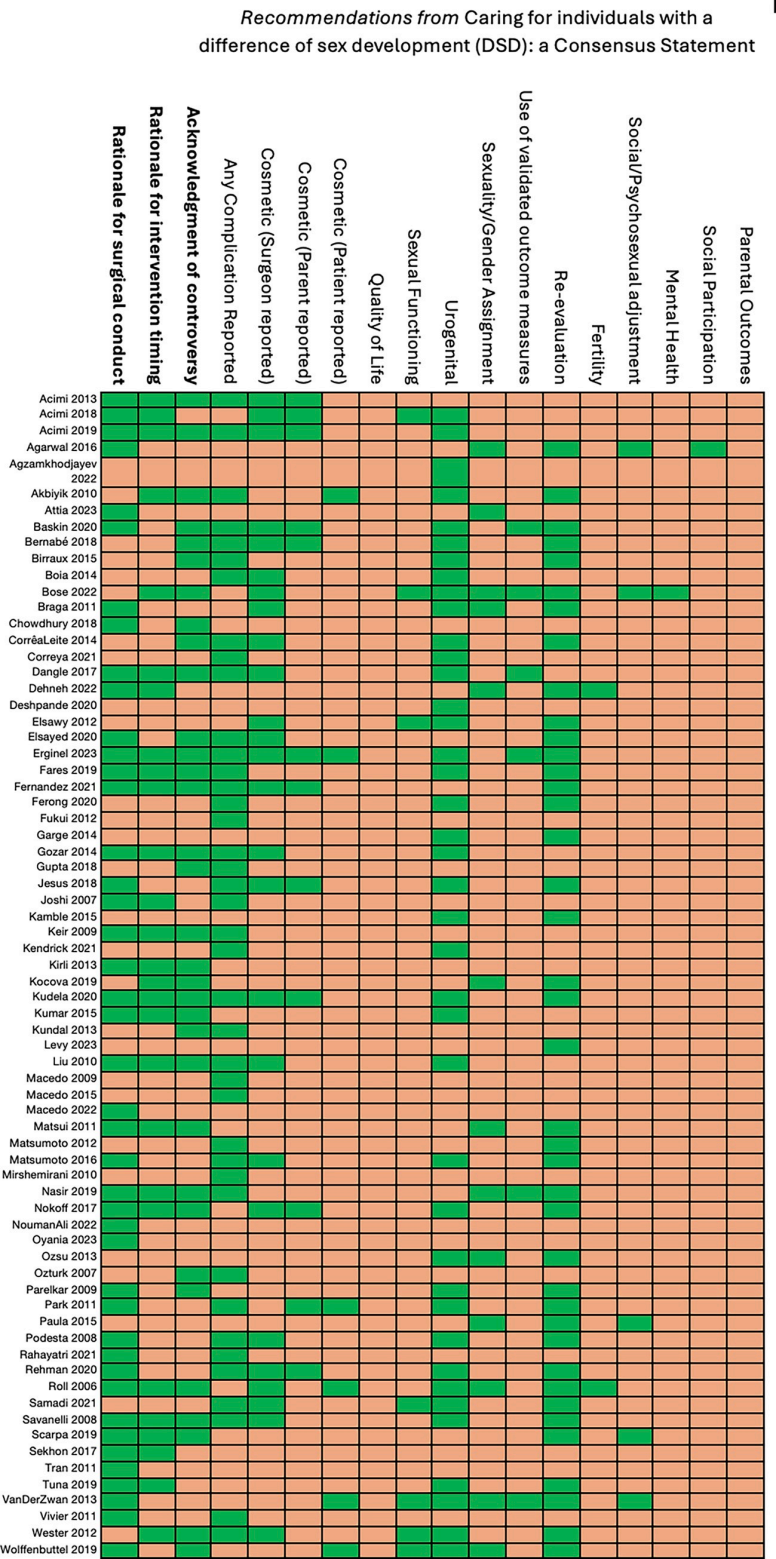
Chart reporting the inclusion of rationales and acknowledgment of controversy in included articles, as well as a tabulation of outcomes from and characteristics of included studies, in line with the recommendations from caring for individuals with a difference of sex development [2].

*Fig 2* is a representation of the outcomes reported in included articles, categorised in line with the recommended reporting outcomes in the 2018 publication *Caring for Individuals with a Difference of Sex Development* [2]. *Fig* 2 also indicates which articles reported rationales for the conduct or timing of surgery, and those that acknowledged controversy. The most common outcomes collected in included literature were cosmetic outcomes, primarily reported by surgeons or parents, complication rates and urogenital outcomes. There was a notable lack of validated measures used by studies when assessing any surgical outcome, particularly cosmetic, and no studies collected any data on child quality of life or parental outcomes (i.e., parental mental health).

### Rationales provided for conducting “sex-normalising” procedures and their timings

Four codes were inductively identified to categorise the rationales for “sex-normalising” procedures: Anatomical/Functional/Cosmetic (used in 50.7% of studies for either conduct or timing of intervention; n = 36), Perceived Goal of Aligning with Assigned Sex (39.4%; n = 28), Parental Desire (19.7%; n = 14), Belief of Best Practice (9.9%; n = 7) and Access (1.4%; n = 1). 33.8% of studies provided no rationale for the timing or conduct of “sex-normalising” interventions (n = 24).

#### Anatomical/functional/cosmetic

Studies providing anatomical/functional/cosmetic reasons as a rationale for the timing or conduct of procedures investigated CAH (n = 17), diphallia, MGD (n = 3, respectively), PMDS, MRKH syndrome, Herlyn-Werner-Wunderlich syndrome, unspecified DSDs, PAIS (n = 2, respectively), genital ambiguity with high vaginal confluence, true hermaphroditism, genital ambiguity, 5-alpha reductase deficiency, ovotesticular syndrome, ovotesticular DSD, perineal hypospadias in DSD, persistent cloaca and 46, XX DSD (n = 1, respectively).

*Rationale for conducting procedure*. Cosmetic rationalisations for intervention were primarily based around the goal of achieving a ‘normal’ external genital appearance, corresponding with the sex proposed for children by the surgical team or parents [5–8, 39–57]. This was sometimes done through critically presenting ambiguous genitalia, labelling them as ‘ugly’ [44] or something to ‘correct’ [53]. Cosmetic goals were generally unspecific, with authors striving for cosmetic results that were ‘satisfactory’ [54, 57], ‘aesthetically pleasing’ [49], ‘normal’ [8, 50, 51], ‘good’ [52], ‘natural-looking’ [47], ‘favourable’ [55] or ‘typical’ [56]. Improved cosmesis, determined primarily by parents or surgeons (*Fig 2*), was perceived by surgical teams to be linked to improved psychosocial outcomes such as anticipated improved parent-child attachment, reduced stigma or psychological distress, or perceived improvements in developments of gender identity [6, 43].

Anatomically, surgical interventions were proposed as a way to prevent perceived incorrect virilisation of children and to align genitalia with the perceived ‘typical’ anatomy of the sex proposed by parents or surgeons [5, 7, 43, 58, 59]. Studies often articulated this by proposing interventions as a way of resituating anatomical structures to their ‘typical’ position [54, 60]. As with cosmetic outcomes, goals were unspecific [61], with some teams describing the results they were striving for as ‘satisfactory’ [8, 49, 50, 62]. Preventing downstream complications associated with ambiguous genitalia, such as endometriosis in Herlyn-Werner-Wunderlich Syndrome [63], was also cited as anatomical rationale for intervention conduct. Anatomical justifications were also used to select and justify the type of procedure being offered, such as one surgical team electing for ‘conservative’ approaches to minimise tissue removal for children who do not yet have definitive gender identity [64]. There was a notable impression by some authors that anatomical goals were secondary to aforementioned cosmetic rationales [50].

Many functional rationales were unspecific and subjective, including achieving ‘satisfactory’ [57], ‘good’ [8, 52], ‘proper’ [40], ‘typical’ [56], ‘favourable’ [55] or ‘desirable’ [53] functional outcomes. More specific functional goals included achieving sexual function, including the prospect of ‘successful’ future intercourse (with the implication that sexual intercourse constituted penis-in-vagina, penetrative sex) [42, 45, 48] and achieving sexual arousal [5, 41, 44, 50], menstruation [5, 42, 43] and protection of fertility [5, 62]. Follow-up times for studies were often not long enough to be able to reliably confirm these functional goals (*Table 1*). One study suggested a recent shift in prioritisation by global surgical teams towards protecting sexual satisfaction and sensate genital tissues in interventions [44]. There were also notable instances in which authors recognised uncertainty surrounding the functional impact of interventions, with articles suggesting post-intervention genitalia may be ‘more natural looking, and *possibly* functioning [emphasis added]’ [47] and that interventions ‘*may* facilitate sexual intercourse [emphasis added]’ [45]. Similar to anatomic rationales, some authors implied functional outcomes were secondary to the cosmetic results they desired [46, 50].

*Rationale for timing of procedure*. Cosmetic rationalisations for early intervention were often identical to those justifying the conduct of procedures, with no clear distinction articulated on the relative benefits of conducting these interventions in infancy. Some studies argued that the long-term psychological impact of the appearance of ambiguous genitalia is greater if interventions are not conducted early, without citing any recommendations or wider literature justifying this [6, 39]. Other studies suggested that better compliance from patients and a lack of recollection in adulthood should justify early conduct of interventions [55]. Anatomical justifications for early surgery were more detailed, centring around the argument that early conduct is associated with better surgical outcomes. Several studies suggested that increased oestrogen exposure in the first month of life will provide better vascularised tissue and thicker walls of vaginal tissue [7, 55], with other studies providing alternate developmental anatomical justifications, such as undeveloped penile circumflex arteries in infants leading to reduced risks of intra-operational bleeding (an uncited justification) [44]. Early age was also suggested to be the most appropriate time for preventing a range of complications that could have life-long effects [61, 63, 65, 66]. Studies also suggested general higher success rates of operations conducted in infancy, without expanding on the specificities of this [67]. “Sex-normalising” interventions in infancy were also rationalised in infants requiring multiple surgeries. For example, one study suggested the benefits of concurrently performing vaginal reconstruction in a child with MRKH syndrome who incidentally had an anorectal malformation, with the justification that scarring of the urinary tract and rectum from anorectal reconstruction in this case may make vaginoplasty more difficult in later life [68]. Early intervention was also suggested as a way to prevent anticipated incorrect virilisation and puberty associated with retained gonads, the justifications for which were uncited [62, 69]. Interestingly, one included study initially promoting early surgical intervention changed their mind following their study, moving to advocating for delaying intervention until puberty, with the anatomical justification that post-pubertal genital skin is more robust than the introital skin of infants [7]. Functional justifications for early intervention centred around the fact that early surgery will more effectively achieve desired function outcomes [61], including maintaining reproductive potential [63] and metabolic development [62].

#### Perceived goal of aligning with assigned sex

The 28 studies reporting a perceived goal of aligning with an assigned sex as a rationale for the timing or conduct of procedures investigated CAH (n = 14), ovotesticular DSD (n = 4), 5-alpha reductase deficiency, MGD (n = 2, respectively), MRKH syndrome, true hermaphroditism, unspecified DSDs, ambiguous genitalia and 46, XX DSD (n = 1, respectively).

*Rationale for conducting procedure*. Rationalising procedures through pursuing a binary assigned sex for infants manifested in several ways. Anatomically, articles cited an intention to use interventions to create ‘normal’ external genitalia corresponding with the assigned sex of the infant by parents or surgeons [7, 8, 40, 42–44, 70, 71] or to remove genitals unconcordant with the sex assigned to infants [7, 65, 70–73]. Some articles went further than this and identified interventions as a way to *manifest* the assigned sex (and subsequently, gender identity) chosen for the infant [5, 74], suggesting interventions could ‘*create* anatomical […] female gender [emphasis added]’ [58] or ‘*convert* to the male sex [emphasis added]’ [75]. Facilitating psychosocial adjustment through conforming to a binary sex was also cited as an ethical motivation for conducting interventions [43, 45, 76], based on the assumption that genital appearance influences the development of the sexual psyche [39]. Teams also suggested that intervention could achieve certain sexual orientations and sexual development associated with the assigned sex [62], with one study citing hysterectomy and vaginectomy in CAH patients as a way to provide ‘satisfactory male gender identity and heterosexual orientation’ [62], and another suggesting intervention would facilitate the infant becoming a ‘functional member of the society’ [77].

*Rationale for timing of procedure*. The key rationalisation of early intervention timing when enacting the perceived goal of aligning with an assigned sex was the assumption that earlier intervention will more effectively facilitate gender identity development in line with sex assigned to a child by parents or surgeons, and limit psychological distress associated with an ambiguous sex [5, 6, 41, 42, 46, 62, 70, 74]. One study extends this to suggest that early intervention will facilitate healthy body image development, a suggestion that did not cite any recommendations or relevant literature [39]. Some rationales relied on a belief that gender identity formation occurs in early life and can be strongly influenced by environmental surroundings [39, 78]. Others cited early intervention as key for preventing psychological distress, without detailing the specific reason or evidence-base as to why *early* intervention is protective against this [8, 44]. One study cites early intervention as an important tool to prevent societal barriers related to assigned sex for people with congenital variations in sex characteristics, specifically highlighting the complicated sports life in female athletes with 5-alpha reductase deficiency [79].

#### Parental desire

The 14 studies reporting parental desire as a rationale for the timing or conduct of procedures investigated CAH (n = 10), ovotesticular DSD (n = 3) and 5-alpha reductase deficiency (n = 1). Both anticipated (n = 11) and vocalised parental desire (n = 4) were used as rationales.

*Rationale for conducting procedure*. Several articles predicted that external genital appearance that did not match conventional conceptions of male or female bodies would increase parental anxiety, distress and embarrassment, and used mitigating this as a justification for intervention [7]. One study specifically identifies the anticipated parental embarrassment surrounding a large clitoris in a child with CAH as a rationale for intervention [39]. Articles also assumed that parental acceptance and parent-child attachment would be improved through surgical intervention, despite often citing no relevant literature or discussions with family to justify this [6]. Surgical teams also articulated a difficulty in navigating voiced parental distress. Two surgical teams report going against their own recommendation to delay treatment due to intense parental desire for early intervention [40, 78]. Parental desire was not only expressed for surgical intervention, but also for the assigned sex of their child. One study reported a strong parental preference to raise their child with ovotesticular DSD as a girl, which was then used as a rationale for conducting reconstructive vaginoplasty [80].

*Rationale for timing of procedure*. Some articles anticipated that specifically *early* reconstruction would alleviate anticipated psychological distress in parents [7, 8, 41], improve parental attachment [69, 70] and increase parental comfort [62]. One study cites parents asking for early orchiopexy for their child with ovotesticular DSD before they began school to ensure wellbeing within the family, school and wider society [71]. In some cases, authors acknowledged that conducting early intervention was going against current global trends towards delaying intervention, but used strong parental desire in their local context to justify this. For example, one study cited parental anxiety specifically resulting from their sociocultural context as reason to complete the procedure as soon as possible [69]. Ethical justifications relating to preventing parental harm were also used to justify early intervention, with one study stating the birth of a child with ambiguous genitalia is a ‘tragedy’ for parents and thus ‘prolonging this suffering’ is morally unjustifiable [81].

#### Belief of best practice

The 7 studies reporting belief of best practice as a rationale investigated CAH (n = 5), unspecified DSDs and ambiguous genitalia (n = 1, respectively).

*Rationale for conducting procedure*. All included studies citing clinical documentation to justify interventions cite the 2006 publication, *Consensus Statement on Management of Intersex Disorders* [9], referencing its recommendations to conduct surgical interventions in cases of severe virilisation, with the goal of not only improving cosmesis but also future sexual function [6, 41, 54, 67].

*Rationale for timing of procedure*. Studies similarly cited the 2006 publication, *Consensus Statement on Management of Intersex Disorders* [9] when justifying early intervention in cases of severe virilisation [6, 41], as well as citing clinical practice guidelines from the Endocrine Society [40, 43, 67, 82] and recommendations from a range of government health authorities [45, 83].

#### Access

One study from the UK investigating CAH reported a logistic issue justifying the timing of infant genital surgery [78]. Despite teams advocating delayed intervention to parents, an imminent move by the family to their country of origin led the clinical team to elect to conduct the intervention in their specialist unit rather than risk the intervention being conducted in non-specialist facilities at a later date.

#### No rationale

44 studies provided no rationale for timing of surgery, 28 no rationale for the conduct of surgery, and 24 studies no rationale for either. These studies providing no rationale for either timing or conduct investigated diphallia (n = 8), PMDS, ovotesticular DSD (n = 4, respectively), CAH, transverse testicular ectopia(n = 3, respectively), gonadal dysgenesis (n = 2), 5-alpha reductase deficiency, 45,X/46, XY DSD, Antley-Bixler syndrome, PAIS, severe hypospadias and microphallus, sex chromosome mosaicism and Herlyn-Weber-Wunderlich syndrome (n = 1, respectively)

### Acknowledgement of controversy

47.9% of studies (n = 34) acknowledged controversy surrounding the interventions they were reporting. Three codes were developed to describe studies’ acknowledgment of the controversy surrounding “sex-normalising” surgical interventions: controversy surrounding the quality and standards of care (n = 29), controversy surrounding the cultural, gender and social well-being implications of interventions (n = 18) and controversy surrounding the ethics of the procedure (n = 15).

#### Standards of care and quality

There was extensive debate in included studies addressing concerns about the quality of interventions, specifically highlighting a lack of information about long-term outcomes of the surgery [42, 43, 55, 60, 64, 84], harms caused by the surgery [6, 13, 40, 43, 56, 84], lack of evidence on the number of operations required [7, 39, 43] and contention about intervention timing [7, 8, 39, 40, 42, 43, 53, 55, 62, 64, 68, 71, 74, 76, 81]. Studies navigated these controversies in different ways. Some authors introduced the ethical debate surrounding interventions, and went on to dismiss the validity of these concerns and continue with the intervention regardless [60, 62, 68, 85]. Several studies also explored concerns about intervention quality, but continued with various levels of accommodations for these controversies, including leaving surgical decision-making up to expertise of the surgical team [7, 55], changing surgical procedure [5, 40, 42, 43, 53, 64, 81, 86], individualising care [87] or providing psychological support to parents and children [71, 75].

#### Culture/gender/social well-being

Studies that addressed controversies surrounding the cultural, gender and social considerations of interventions introduced this debate through discussing the age of gender identity and sexual development, largely suggesting that by 2–3 years of age, children may be able to correctly label themselves of a certain gender, thus justifying intervention before this age [39, 78]. Some studies recognised that controversies surrounding sex determination were increasing [7], particularly with concerns around higher prevalence of gender dysphoria among individuals with congenital variations in sex characteristics due to assignment of sex not matching future gender identity [42, 64, 69]. Often these controversies were dismissed through authors citing previous experience or small-scale qualitative evidence citing low levels of gender-related regret in adults having undergone infant genital surgery [64, 78]. The rights of the child and perceived rights of parents also pervaded in these debates, with authors debating the gender preferences of families and the future gender identity of individuals with congenital variations in sex characteristics [46, 71, 73, 75]. In addition to the aforementioned ways of navigating these controversies, such as offering patient-centred, multidisciplinary, individualised care for patients [7, 46, 64, 71], studies also cited the possibility of a sex reversal later in life to rationalise controversial decisions to undergo procedures [71, 74, 79] or recommended psychological support for children who develop gender dysphoria from incorrect sex assignment from surgeons or parents post-intervention [69, 75]. Authors also justified intervention in the face of these controversies by suggesting conservative surgery could balance future risks of gender dysphoria while protecting children from growing up in non-accepting environments [64, 71].

#### Ethics

A range of ethical controversies were acknowledged by included studies, primarily concerning the ability to provide informed consent for an irreversible intervention in infancy, and the interventions’ implied impact on cosmesis and gender identity [40, 42, 53, 64, 69, 78]. Some studies further extend this debate to discuss the relative rights of the individual and the perceived rights of parents/guardians to request such intervention [46, 55, 74, 81]. There is a recognition in some articles about a changing trend in the ethical approach to “sex-normalising” interventions, suggesting a global trend towards prioritising rights of the individual and postponing interventions [46, 71]. These ethical controversies were navigated in different ways. Often ethical concerns were considered but not explored or integrated into decision-making [86, 88]. The aforementioned surgical, functional, and anatomical rationales are also used by authors as considerations that overwhelm the ethical concerns of interventions, despite many concurrently noting that evidence on the relative efficacies of early and late surgery are lacking [40, 53, 55, 74]. Notably, one team changed their recommendations to promote later intervention after their study, citing ethical concerns arising when considering high complication rates and low patient satisfaction with early intervention [42]. Placement of the global ethical debate within the context teams were working was also used to rationalise why teams found it appropriate to continue to conduct interventions, primarily suggesting that perceived rights of parents overwhelm the rights of the child in certain settings [46, 69, 74, 75]. Other studies introduced ethical controversies surrounding interventions but rationalised their conduct through providing individualised care to patients from a multidisciplinary team and providing psychological counselling to those involved [69, 71]. Some studies adapted their interventions based off of ethical concerns about irreversibility [64], with authors of one diphallia study promoting early surgery using a technique that did not remove penile tissue as a way to (in their view) address ethical concerns surrounding irreversibility [86]. One study adapted their study to focus on vaginoplasty rather than clitoroplasty in their report on a child with CAH to incorporate ethical concerns, due to vaginoplasty being ‘less debated’ in their setting [89].

## Discussion

This manuscript reports the findings of the first systematic review investigating the rationales, outcomes and controversies surrounding elective, “sex-normalising” interventions for infants with congenital variations in sex characteristics. Included articles were largely case reports, matching previous observations about the extant literature [2, 4]. Among a geographically diverse set of articles, we noted outcome reporting that did not align with recommended reporting guidance. Rationales reported by surgical teams both concerning the conduct of interventions and their timings were heterogeneous across included studies, but broadly included anatomical/cosmetic/functional rationales, a perceived desire of aligning with a sex assigned by surgeons and parents, parental desire, and a belief of best practice. Studies acknowledged controversies surrounding the quality of interventions, cultural, gender and social considerations, and the ethics surrounding surgeries, while demonstrating different ways of integrating these controversies into their rationale for the continued justification of surgical intervention. 62.0% of studies reported no specific rationale for intervention timing, 39.4% reported no rationale for conduct and 52.1% acknowledged no controversy in the interventions they were conducting, implying that in some settings these surgical interventions remain accepted, common practice.

Cosmesis emerged as an important rationale for the conduct of interventions, as well as being one of the most commonly reported outcome measures collected in included articles. There was notable subjectivity in the reporting of cosmesis, with subjective, descriptive terminology used by surgeons and parents to determine successful cosmetic outcomes, combined with a lack of validated measures used for outcome appraisal. There is no guarantee that infant genital surgery will improve long-term cosmetic outcomes for the individual concerned; indeed, some adults with congenital variations in sex characteristics who have received surgical intervention in infancy report dissatisfaction and distress associated with their post-surgical genital appearance [90], concerns which will sometimes require multiple repeat surgeries to address [91]. Decisions about genital cosmesis should not be made without the active participation and consent of the individual to whom the genitalia belong, and thus elective, irreversible genital surgical interventions for infants and children with congenital variations in sex characteristics should not take place before the individual can decide for themselves whether they would like to undergo such procedures [92].

19.7% of studies reported parental distress as rationale for either the conduct or timing of interventions, primarily citing an anticipated or voiced parental desire for improved genital cosmesis or for genitals that align more with those associated with ‘typical’ male or female bodies. Medical teams also suggested surgery could relieve psychological distress in parents, improve parental attachment and increase parental comfort. Importantly, a belief that infant surgery was not best practice by surgical teams was occasionally overwhelmed by intense parental desire for intervention in infancy or early childhood, indicating the respect given to parental desire in many settings. Medical teams in some settings struggled with balancing clearly voiced parental desire for early intervention against medical recommendations and human rights imperatives to delay decision-making surrounding intervention until children with congenital variations in sex characteristics are able to participate in decision-making processes. This underscores the need for multidisciplinary teams supporting infants and children with congenital variations in sex characteristics and their parents. Medical teams have a critical role to play in guiding and managing ethical decision-making with parents in clinical settings and sociocultural environments where the rights of the child and obligations of the parents to make decisions which benefit the child do not necessarily match parental desire [93]. Known drivers of parental distress should be better and consistently integrated into parent and family counselling and psychosocial support, and delivered by qualified professionals like psychologists and social workers [4]. This counselling may include, for example, a focus on destigmatising congenital variations in sex characteristics, providing clear supportive pathways for communicating with their child on their variation as they grow, being candid on the risks of early surgery for a child, and connecting parents to the intersex community and parents of children with congenital variations in sex characteristics.

39.4% of studies reported a perceived goal of aligning with an assigned sex from parents or surgeons as a rationale for intervention conduct or timing, with arguments around promoting a specific gender also pervading into cosmetic, functional, and anatomical rationales. These rationales largely rely on the assumption that early life experience, including cosmetic, social, and functional features, predicts future gender identity; a theory cited by several studies when navigating sex assignment controversies [39, 78]. This is reminiscent of mid-20th century arguments about gender identity development, popularized by John Money (the work of whom is cited by some included articles [42, 81] which suggests gender identity is socially constructed in childhood and thus can be re-assigned in infancy, with external factors such as social environment and anatomical features made to match this chosen assignment [94]. Although this theory is now debunked [95, 96], it still pervades in rationales for intervention conduct and a belief that earlier intervention is more efficacious at promoting gender identity development aligned with an assigned sex from parents or medical teams. There is no evidence to suggest surgical intervention for infants with congenital variations in sex characteristics is associated with ‘better’ gender identity development. In fact, several studies note an increased prevalence of gender dysphoria in individuals having undergone “sex-normalising” surgical intervention in infancy [2, 97, 98]. Irreversible, elective genital surgical interventions with the rationale of promoting the development of a specific gender identity are unethical without the full, free, and informed consent and decision-making of the person concerned. Rather, counselling and support for children and parents should be provided throughout infancy, childhood and adolescence, until an individual is old enough to decide for themselves whether they wish to undergo such interventions, and, if they do desire intervention, are able to understand and provide informed consent for any desired interventions.

Forty-six studies were excluded from analysis after full-text screening for citing malignancy as a rationale for conducting interventions, as we determined mitigation of a perceived malignancy risk to be sufficient to consider an intervention to be non-elective. The importance of mitigating the risk of childhood malignancy should not be understated. However, it is important to ensure that the choice to undertake prophylactic genital surgeries (versus a more conservative course of biopsy and monitoring) before a child can provide full, free and informed consent and participate in decision-making processes is done with caution, and that any risk of malignancy is justified and evidence-based. Twenty-six of these forty-six studies cited malignancy as a rationale for surgery for a congenital variation in sex characteristics for which surgical intervention was *not* recommended as a malignancy mitigation strategy in the 2006 publication, *Consensus Statement on Management of Intersex Disorders* [9], which was the last guidance to segregate malignancy risk by individual congenital variation in sex characteristics. This echoes a lack of clarity on the malignant potential of different congenital variations in sex characteristics, which is noted in the extant literature [36, 37, 99]. The expertise of this team did not allow for a critical appraisal of the validity of oncological concerns justifying interventions. Future research should focus on fully elucidating causal pathways and epidemiological connections between individual congenital variations in sex characteristics and malignancy to ensure irreversible genital surgeries are not being conducted when biopsy and monitoring may be sufficient malignancy mitigation strategies.

The social and cultural complexity of congenital variations in sex characteristics across different geographic settings and demographic groups was cited as a reason for conducting interventions and permeated throughout discussions about intervention controversy. This was of particular importance when considering parental desire and concerns about raising a child in a setting that could potentially be stigmatising. Attitudes towards people with congenital variations in sex characteristics differ largely across settings, with cultural considerations about gender preference, identity and stigma towards children with congenital variations in sex characteristics driving strong parental and surgeon desire for intervention in certain settings [69, 100]. Concerns about raising a child with congenital variations in sex characteristics should be acknowledged and integrated into discussions with parents (for example, by providing resources, including through peer support from intersex persons and their parents, on how to communicate with other family and community members). Parental or surgeon concerns, however, should not be used to justify the conduct of elective, irreversible genital surgeries before an individual can provide full, free and informed consent.

International human rights treaty bodies have recommended the prohibition of irreversible, elective, genital surgeries conducted on children with congenital variations in sex characteristics before they can participate in decision-making and give full, free and informed consent [25]. Despite this, we identified that interventions continue to be conducted, based largely around heterogeneous anatomical and functional goals that were not adequately supported by recommendations and the extant medical literature, a desire from parents and surgeons to match genital cosmesis with that typically ascribed to male and female bodies, and a parental desire for intervention conduct. In-keeping with the recommendations of human rights bodies, we recommend that human rights, including the rights of the child, should be protected and prioritised by health systems, and, as such, desire for intervention from parents or surgical teams is not sufficient to justify conducting irreversible, elective, genital interventions before a child can decide for themselves if they want or do not want such procedures, and if they do, provide informed consent to any intervention.

There are notable limitations to this review. Our findings are not entirely representative of the diversity of persons with congenital variations in sex characteristics, given the notable discrepancy in the categorisation of these variations across different disciplines and countries. Our conservative approach to study inclusion, such as excluding studies citing generic urological functional goals and all studies citing malignancy regardless of recommendations from oncological literature, may have led to the exclusion of relevant studies. However, a *strength* of this review is that this conservative approach ensured that all included interventions were clearly elective. There is also likely to be a publication bias towards congenital variations in sex characteristics that aren’t commonly occurring, given the predominance of case reports emerging from our inclusion criteria (i.e., the large number of diphallia cases included, when the variation itself is reported to be limited to one in every five million live births [101]). A decision to exclude articles in which it was not certain if all individuals were under the age of 10, to ensure our findings represented only interventions conducted in infancy and childhood, led to the exclusion of cohort and larger scale studies where these data were not disaggregated by age, potentially biasing the study towards primarily considering lower-quality case reports. Available clinical data lacks adequate sample sizes, independence and relevant control groups [28], undermining the generalisability of the outcomes reported in this review. Although some guidance recommend upscaling longitudinal (including retrospective) outcome assessment studies to address this lack of data [2, 9], it is important that any long-term outcome data is collected from individuals who provided full, free and informed consent to undergo elective, irreversible, genital interventions and to being a participant in any research. Outcome measurements and research priorities should focus on understanding the perspectives, health and wellbeing of the individuals affected and how health services can better meet their current and future needs.

## Conclusion

International human rights monitoring and accountability mechanisms call for the deferral of decision-making around elective, irreversible, “sex-normalising” genital surgeries for infants and children with congenital variations in sex characteristics until individuals can participate in decision-making and provide free, full and informed consent to any desired interventions. This review has identified a heterogenous literature reporting the surgical outcomes, rationales, and controversies of surgical interventions, with notably inconsistent study designs, methods, and outcome reporting. Assessment of surgical rationale and outcomes has revealed that medical teams continue to conduct these interventions in the face of controversy due to desires to mitigate parental distress surrounding the perceived difficulties of raising a child with a congenital variation in sex characteristics, as well as a variety of under-researched or outdated beliefs that doing so mitigates suboptimal anatomical, cosmetic, physiological, and psychological outcomes for a child, or that surgical intervention is best practice.

Irreversible, elective, “sex-normalising” genital interventions on infants or children with congenital variations of sex characteristics are unethical without the full, free, and informed consent and active participation in decision-making of the individual concerned. Rationales of achieving a cosmetic outcome perceived as satisfactory by individuals to whom the genitalia do not belong, matching parental desire or alleviating parental distress, or promoting a specific gender identity do not justify continuation of these procedures.

National legislating and medical regulatory bodies, in collaboration with relevant international and regional organisations, should enhance efforts to end the conduct of irreversible, elective, “sex-normalising” interventions conducted without the full, free, and informed consent of the person concerned, in an effort to promote and protect the right to the highest attainable standard of physical and mental health for people with congenital variations in sex characteristics.

## Supporting information

S1 Text PRISMA checklist. (DOCX)

S2 Text Search strategy. (DOCX)

S1 Table Quality assessment table. (DOCX)

S2 Table Articles excluded at full text with exclusion reason. (CSV)

S3 Table Study characteristics with information on data extraction. (DOCX)
