# Supplementary material for: Perspectives on conducting “sex-normalising” intersex surgeries conducted in infancy: A systematic review
Source: PLOS Glob Public Health. 2024 Aug 28;4(8):e0003568. doi: 10.1371/journal.pgph.0003568 (PMC11356455; doi:10.1371/journal.pgph.0003568)
Supplement: S1 Text — (DOCX) [file pgph.0003568.s001.docx]

**S1 Text – Search Strategy**

*Search Results Summary*

| Database | Results | Date |
| --- | --- | --- |
| Pubmed | 4634 | 30.06.2023 |
| Embase | 4923 | 30.06.2023 |
| Cinahl | 1485 | 30.06.2023 |
| Total | 11042 |  |
| Duplicates | 4079 |  |
| To screen | 6963 |  |

*Pubmed*

| Database: Pubmed | | |
| --- | --- | --- |
| URL: https://pubmed.ncbi.nlm.nih.gov/advanced/ | | |
| Date Searched: 27.06.2023 | | |
| **#** | **Searches** | **Results** |
| 1 | "Disorders of Sex Development"[Mesh] OR "Intersex Persons"[Mesh] OR intersex*[tiab] OR "sex development disorder"[tiab:~3] OR "sex development disorders"[tiab:~3] OR "sex development difference"[tiab:~3] OR "sex development differences"[tiab:~3] OR "sex development disturbance"[tiab:~3] OR "sex development disturbances"[tiab:~3] OR "sexual development disorder"[tiab:~3] OR "sexual development disorders"[tiab:~3] OR "sexual development difference"[tiab:~3] OR "sexual development differences"[tiab:~3] OR "sexual development disturbance"[tiab:~3]OR "sexual development disturbances"[tiab:~3] OR "sex differentiation disorder"[tiab:~3] OR "sex differentiation disorders"[tiab:~3] OR "sex differentiation difference"[tiab:~3] OR "sex differentiation differences"[tiab:~3] OR "sex differentiation disturbance"[tiab:~3] OR "sex differentiation disturbances"[tiab:~3] OR "sexual differentiation disorder"[tiab:~3] OR "sexual differentiation disorders"[tiab:~3] OR "sexual differentiation difference"[tiab:~3] OR "sexual differentiation differences"[tiab:~3] OR "sexual differentiation disturbance"[tiab:~3] OR "sexual differentiation disturbances"[tiab:~3] | 39,990 |
| 2 | ("5-alpha-reductase deficiency"[tiab:~3] OR "5alpha-reductase deficiency"[tiab:~3] OR "5α-reductase deficiency"[tiab:~3]) | 424 |
| 3 | (((ambig*) AND (genital*))) | 2,202 |
| 4 | androgen-insensitivity[tiab] OR feminizing-testicular-syndrome[tiab] OR morris-syndrome[tiab] OR testicular-feminisation[tiab] OR testicular-feminiz*[tiab] OR testis-feminization[tiab] | 2,651 |
| 5 | aromatase-deficiency[tiab] | 178 |
| 6 | clitoromegaly[tiab] | 385 |
| 7 | adreno-genital-syndrome[tiab] OR androgenital-syndrome[tiab] OR adrenogenital-disorder*[tiab] OR adreno-genital-disorder*[tiab] OR congenital-adrenal-gland-hyperplasia[tiab] OR congenital-adrenal-virili*[tiab] OR congenital-adrenocortical-hyperplasia[tiab] OR congenital-adrenogenital-syndrome[tiab] OR debre-fibiger-syndrome[tiab] OR congenital-adrenal-hyperplasia[tiab] | 5,823 |
| 8 | diphallus[tiab] OR double-penis[tiab] OR penile-duplication[tiab] OR supernumerary-penis[tiab] OR diphallia[tiab] | 168 |
| 9 | feminization-syndrome[tiab] OR male-feminization[tiab] OR pathological-feminization[tiab] | 321 |
| 10 | freemartin-syndrome[tiab] OR freemartinism[tiab] | 87 |
| 11 | gonadal-dysgenesis[tiab] OR gonad-dysgenesis[tiab] OR turner-syndrome[tiab] OR Ullrich-syndrome[tiab] OR ulrich-syndrome[tiab] OR XO-syndrome[tiab] OR X-chromosome-monosomy[tiab] OR testicular-DSD*[tiab] OR De-la-Chapelle-syndrome*[tiab] OR frasier-syndrome*[tiab] OR Kallmann-syndrome*[tiab] OR WAGR-syndrome*[tiab] OR Drash-syndrome*[tiab] | 8,733 |
| 12 | hermaphrodit*[tiab] OR psuedohermaphrodit*[tiab] | 6,516 |
| 13 | Leydig-cell-agenesis[tiab] OR Leydig-cell-aplasia[tiab] OR Leydig-cell-hypoplasia[tiab] | 97 |
| 14 | microphallus[tiab] OR micropenis[tiab] OR micro-penis[tiab] | 1,085 |
| 15 | sexual-infantilism[tiab] OR genital-infantilism[tiab] | 168 |
| 16 | didelphic-uterus[tiab] OR didelphys-uterus[tiab] OR double-uterus[tiab] OR uterine-didelphys[tiab] OR uterus-didelphis[tiab] OR uterus-duplex[tiab] OR uterus-duplication[tiab] OR uterus-dydelphys[tiab] OR uterus-reduplication[tiab] OR uterus-didelphys[tiab] | 963 |
| 17 | pathological-androgenization[tiab] OR pathological-masculinisation[tiab] OR pathological-masculinization[tiab] OR virilized-women[tiab] OR virilizing-syndrome[tiab] OR virilization[tiab] | 2,108 |
| 18 | "Hypospadias"[Mesh] OR hypospadia*[tiab] | 8,213 |
| 19 | Mayer-Rokitansky-Küster-Hauser-Syndrome*[tiab] OR MRKH-Syndrome*[tiab] OR Persistant-Mullerian-duct-syndrome*[tiab] OR PMD-Syndrome*[tiab] | 850 |
| 20 | "Surgical Procedures, Operative"[Mesh] OR "General Surgery"[Mesh] OR "surgery"[Subheading] OR postoperat*[tiab] OR operat*[tiab] OR removal[tiab] OR refashion*[tiab] OR excision*[tiab] OR electroexcision*[tiab] OR surg*[tiab] OR electrosurg*[tiab] OR reconstruct*[tiab] OR repair*[tiab] OR resection*[tiab] OR ectomy[tiab] OR ectomies[tiab] OR urethroplast*[tiab] OR clitoroplast*[tiab] OR phalloplast*[tiab] OR orchiopex*[tiab] OR perineoplast*[tiab] OR Vulvoperineoplast*[tiab] OR ethic*[tiab] | 6,630,101 |
| 21 | #1 OR #2 OR #3 OR #4 OR #5 OR #6 OR #7 OR #8 OR #9 OR #10 OR #11 OR #12 OR #13 OR #14 OR #15 OR #16 OR #17 OR #18 OR #19 | 59,493 |
| 22 | #20 AND #21 | 14,273 |
| 23 | ("Animals"[Mesh] NOT ("Animals"[Mesh] AND "Humans"[Mesh])) | 5,133,370 |
| 24 | #22 NOT #23 | 13,477 |
| 25 | "Letter"[Publication Type] OR "Editorial"[Publication Type] OR "Lecture Note"[Publication Type] | 1,874,530 |
| 26 | #24 NOT #25 | 12,908 |
| 27 | (gender-affirm*[ti] OR gender-confirm*[ti] OR (facial[ti] AND femini*[ti]) OR glottoplast*[ti] OR chest-masculini*[ti] OR neck-femini*[ti] OR feminizing-mandibuloplasty[ti] OR vestibular-femini*[ti] OR (femini*[ti] AND frontoorbitoplasty[ti]) OR Parsonage-Turner-syndrome[ti] OR (Transgender*[ti] NOT (Transgender*[ti] AND intersex*[ti])) | 7,132 |
| 28 | #26 NOT #27 | 12,844 |
| 29 | "Child"[mh] OR "infant"[mh] OR "Infant, Newborn"[Mesh] OR "adolescent"[mh] OR "pediatrics"[mh] OR "minors"[mh] OR infan*[tiab] OR newborn*[tiab] OR new-born*[tiab] OR neo-nat*[tiab] OR neonat*[tiab] OR picu*[tiab] OR nicu*[tiab] OR baby[tiab] OR babies[tiab] OR toddler*[tiab] OR child*[tiab] OR adolescen*[tiab] OR pediatric*[tiab] OR paediatric*[tiab] OR preschool*[tiab] OR pre-school*[tiab] OR boy[tiab] OR boys[tiab] OR girl*[tiab] OR kinder[tiab] OR kindergart*[tiab] OR kindergard*[tiab] OR kid[tiab] OR kids[tiab] OR enfant*[tiab] OR nursery[tiab] OR nurseries[tiab] OR creche*[tiab] OR daycare*[tiab] OR youth[tiab] OR young-patient*[tiab] OR student*[tiab] OR pupil*[tiab] OR teen*[tiab] OR pubesc*[tiab] OR prepubesc*[tiab] OR congenital*[tiab] OR born[tiab] OR inborn[tiab] OR puberty[tiab] OR retrospective[tiab] | 5,763,240 |
| 30 | #28 AND #29 | 8,989 |
| 31 | #28 AND #29 Filters: from 2006/7/1 - 2023/6/30 | 4,634 |

*Cinahl*

| Database: Cinahl | | | |
| --- | --- | --- | --- |
| URL: www.ebscohost.com | | | |
| Date Searched: 30.06.2023 | | | |
| **S** | **Searches** | **Results** | **Column1** |
| 1 | (MH "Disorders of Sex Development+") OR (MH "Intersex Persons") OR OR (MH "Pseudointersexuality") OR TX (intersex*) OR (TX (disorder* OR difference* OR divergence* OR disturbance*) N3 (sex-development* OR sex-differentiation* OR sexual-development* OR sexual-differentiation*)) | 3791 |  |
| 2 | (TX (5-alpha-reductase OR 5alpha-reductase OR 5α-reductase) N3 (deficiency)) | 14 |  |
| 3 | (TX (ambig*) N3 (genital*)) | 253 |  |
| 4 | (TX (androgen-insensitivity OR feminizing-testicular-syndrome OR morris-syndrome OR testicular-feminisation OR testicular-feminiz* OR testis-feminization)) | 206 |  |
| 5 | TX (aromatase-deficiency) | 27 |  |
| 6 | TX (clitoromegaly) | 51 |  |
| 7 | TX (adreno-genital-syndrome OR androgenital-syndrome OR adrenogenital-disorder* OR adreno-genital-disorder* OR  congenital-adrenal-gland-hyperplasia OR congenital-adrenal-virili* OR congenital-adrenocortical-hyperplasia OR congenital-adrenogenital-syndrome OR debre-fibiger-syndrome OR congenital-adrenal-hyperplasia) | 763 |  |
| 8 | TX (diphallus OR double-penis OR penile-duplication OR supernumerary-penis OR diphallia) | 22 |  |
| 9 | TX (feminization-syndrome OR male-feminization OR pathological-feminization) | 8 |  |
| 10 | TX (freemartin-syndrome OR freemartinism) | 2 |  |
| 11 | TX (gonadal-dysgenesis OR gonad-dysgenesis OR turner-syndrome OR Ullrich-syndrome OR ulrich-syndrome OR XO-syndrome OR X-chromosome-monosomy OR testicular-DSD* OR De-la-Chapelle-syndrome* OR frasier-syndrome* OR Kallmann-syndrome* OR WAGR-syndrome* OR Drash-syndrome* ) | 1565 |  |
| 12 | TX (hermaphrodit* OR psuedohermaphrodit*) | 69 |  |
| 13 | TX (Leydig-cell-agenesis OR Leydig-cell-aplasia OR Leydig-cell-hypoplasia) | 3 |  |
| 14 | TX (microphallus OR micropenis OR micro-penis) | 84 |  |
| 15 | TX (sexual-infantilism OR genital-infantilism) | 8 |  |
| 16 | TX (didelphic-uterus OR didelphys-uterus OR double-uterus OR uterine-didelphys OR uterus-didelphis OR uterus-duplex OR uterus-duplication OR uterus-dydelphys OR uterus-reduplication OR uterus-didelphys) | 160 |  |
| 17 | TX (pathological-androgenization OR pathological-masculinisation OR pathological-masculinization OR virilized-women OR virilizing-syndrome OR virilization) | 232 |  |
| 18 | (MH "Hypospadias") OR TX (hypospadia*) | 993 |  |
| 19 | TX (Mayer-Rokitansky-Küster-Hauser-Syndrome* OR MRKH-Syndrome* OR Persistant-Mullerian-duct-syndrome* OR PMD-Syndrome*) | 206 |  |
| 20 | (MH "Surgery, Operative+") OR TX (postoperat* OR operat* OR removal OR refashion* OR excision* or electroexcision* or surg* or electrosurg* OR reconstruct* OR repair* OR resection* OR ectomy OR ectomies OR urethroplast* OR clitoroplast* OR phalloplast* OR orchiopex* OR orchidopex* OR perineoplast* OR Vulvoperineoplast* OR ethic*) | 1,688,356 |  |
| 21 | S1 OR S2 OR S3 OR S4 OR S5 OR S6 OR S7 OR S8 OR S9 OR S10 OR S11 OR S12 OR S13 OR S14 OR S15 OR S16 OR S17 OR S18 OR S19 | 6336 |  |
| 22 | S20 AND S21 | 2170 |  |
| 23 | (MH "Animals+") NOT ((MH "Animals+") AND (MH "Human")) | 93963 |  |
| 24 | S22 NOT S23 | 2153 |  |
| 25 | (TI (gender-affirm* OR gender-confirm* OR (facial N3 femini*) OR glottoplast* OR chest-masculini* OR neck-femini* OR feminizing-mandibuloplasty OR vestibular-femini* OR (femini* N2 frontoorbitoplasty) OR Parsonage-Turner-syndrome or Transgender*)) | 5178 |  |
| 26 | S24 NOT S25 | 2101 |  |
| 27 | ((MH "Adolescence+") or (MH "Adolescent Health") OR (MH "Adolescent Medicine") or (MH "Adolescent, Hospitalized") or (MH "Child+") or (MH "Child Health") or (MH "Child, Hospitalized") or (MH "Child, Preschool") or (MH "Infant+") or (MH "Infant, Newborn+") OR (MH "Infant, Premature") or (MH "Neonatology") or (MH "Pediatrics+") OR (TX (infan* or newborn* or new-born* or neo-nat* or neonat* or picu* or nicu* or baby or babies or toddler* or child* or adolescen* or pediatric* or paediatric* or preschool* or pre-school* or boy OR boys or girl* or kinder or kindergart* or kindergard* or kid or kids or enfant* or nursery or nurseries or creche* or daycare* or youth OR young-people OR young-patient* OR student* OR pupil* OR teen* OR pubesc* OR prepubesc* OR congenital* or born or inborn or puberty or restrospective ) ) | 1965539 |  |
| 28 | S26 AND S27 | 1644 |  |
| 29 | S28 AND Published Date: 20060701-20231231 | 1,485 |  |

*Web of Science*

| Database: Web of Science | | | |
| --- | --- | --- | --- |
| URL: | | | |
| Date Searched: Not used | | | |
| **#** | **Searches** | **Results** | **Column1** |
| 1 | TS=(intersex*) OR TS=((disorder* OR difference* OR divergence* OR disturbance*) NEAR/3 (sex-development* OR sex-differentiation* OR sexual-development* OR sexual-differentiation*)) | 9226 |  |
| 2 | TS=((5-alpha-reductase OR 5alpha-reductase OR 5α-reductase) NEAR/3 (deficiency)) | 535 |  |
| 3 | TS=((ambig*) NEAR/3 (genital*)) | 1936 |  |
| 4 | TS=(androgen-insensitivity OR feminizing-testicular-syndrome OR morris-syndrome OR testicular-feminisation OR testicular-feminiz* OR testis-feminization) | 2684 |  |
| 5 | TS=aromatase-deficiency | 520 |  |
| 6 | TS=clitoromegaly | 286 |  |
| 7 | TS=(adreno-genital-syndrome OR androgenital-syndrome OR adrenogenital-disorder* OR adreno-genital-disorder* OR  congenital-adrenal-gland-hyperplasia OR congenital-adrenal-virili* OR congenital-adrenocortical-hyperplasia OR congenital-adrenogenital-syndrome OR debre-fibiger-syndrome OR congenital-adrenal-hyperplasia) | 7552 |  |
| 8 | TS=(diphallus OR double-penis OR penile-duplication OR supernumerary-penis OR diphallia) | 140 |  |
| 9 | TS=(feminization-syndrome OR male-feminization OR pathological-feminization) | 257 |  |
| 10 | TS=(freemartin-syndrome OR freemartinism) | 125 |  |
| 11 | TS=(gonadal-dysgenesis OR gonad-dysgenesis OR turner-syndrome OR Ullrich-syndrome OR ulrich-syndrome OR XO-syndrome OR X-chromosome-monosomy OR testicular-DSD* OR De-la-Chapelle-syndrome* OR frasier-syndrome* OR Kallmann-syndrome* OR WAGR-syndrome* OR Drash-syndrome* ) | 11675 |  |
| 12 | TS=(hermaphrodit* OR psuedohermaphrodit*) | 10759 |  |
| 13 | TS=(Leydig-cell-agenesis OR Leydig-cell-aplasia OR Leydig-cell-hypoplasia) | 145 |  |
| 14 | TS=(microphallus OR micropenis OR micro-penis) | 1145 |  |
| 15 | TS=(sexual-infantilism OR genital-infantilism) | 104 |  |
| 16 | TS=(didelphic-uterus OR didelphys-uterus OR double-uterus OR uterine-didelphys OR uterus-didelphis OR uterus-duplex OR uterus-duplication OR uterus-dydelphys OR uterus-reduplication OR uterus-didelphys) | 737 |  |
| 17 | TS=(pathological-androgenization OR pathological-masculinisation OR pathological-masculinization OR virilized-women OR virilizing-syndrome OR virilization) | 1934 |  |
| 18 | TS=hypospadia* | 7266 |  |
| 19 | TS=(Mayer-Rokitansky-Küster-Hauser-Syndrome* OR MRKH-Syndrome* OR Persistant-Mullerian-duct-syndrome* OR PMD-Syndrome*) | 8305 |  |
| 20 | TS=(postoperat* OR operat* OR removal OR refashion* OR excision* or electroexcision* or surg* or electrosurg* OR reconstruct* OR repair* OR resection* OR ectomy OR ectomies OR urethroplast* OR clitoroplast* OR phalloplast* OR orchiopex* OR orchidopex* OR perineoplast* OR Vulvoperineoplast* OR ethic*) | 7297211 |  |
| 21 | #1 OR #2 OR #3 OR #4 OR #5 OR #6 OR #7 OR #8 OR #9 OR #10 OR #11 OR #12 OR #13 OR #14 OR #15 OR #16 OR #17 OR #18 OR #19 | 56853 |  |
| 22 | #20 AND #21 | 10667 |  |
| 23 | SU=Veterinary Sciences | 766219 |  |
| 24 | #22 NOT #23 | 10302 |  |
| 27 | TI=(gender-affirm* OR gender-confirm* OR (facial NEAR/3 femini*) OR glottoplast* OR chest-masculini* OR neck-femini* OR feminizing-mandibuloplasty OR vestibular-femini* OR (femini* NEAR/2 frontoorbitoplasty) OR Parsonage-Turner-syndrome or (Transgender* NOT (transgender AND intersex))) | 10897 |  |
| 28 | #26 NOT #27 | 10250 |  |
| 29 | TS=(infan* or newborn* or new-born* or neo-nat* or neonat* or picu* or nicu* or baby or babies or toddler* or child* or adolescen* or pediatric* or paediatric* or preschool* or pre-school* or boy OR boys or girl* or kinder or kindergart* or kindergard* or kid or kids or enfant* or nursery or nurseries or creche* or daycare* or youth OR young-people OR young-patient* OR student* OR pupil* OR teen* OR pubesc* OR prepubesc* OR congenital* or born or inborn or puberty or restrospective ) | 5757977 |  |
| 30 | #27 AND #28 | 5741 |  |
