# Supplementary material for: Perspectives on conducting “sex-normalising” intersex surgeries conducted in infancy: A systematic review
Source: PLOS Glob Public Health. 2024 Aug 28;4(8):e0003568. doi: 10.1371/journal.pgph.0003568 (PMC11356455; doi:10.1371/journal.pgph.0003568)
Supplement: S1 Table — (DOCX) [file pgph.0003568.s003.docx]

**S1 Table – Quality Assessment**

*Case Studies (Johanna Briggs Institute Quality Assessment Tool - https://jbi.global/critical-appraisal-tools)*

|  | Were patient's demographic characteristics clearly described? | Was the patient’s history clearly described and presented as a timeline? | Was the current clinical condition of the patient on presentation clearly described? | Were diagnostic tests or assessment methods and the results clearly described? | Was the intervention(s) or treatment procedure(s) clearly described? | Was the post-intervention clinical condition clearly described? | Were adverse events (harms) or unanticipated events identified and described? | Does the case report provide takeaway lessons? |
| --- | --- | --- | --- | --- | --- | --- | --- | --- |
| Agarwal, 2016 | No | No | Yes | Yes | Yes | No | No | No |
| Agzamkhodjayev, 2022 | No | Yes | Yes | Yes | Yes | Yes | No | Yes |
| Attia, 2023 | No | No | Yes | Yes | No | No | No | Yes |
| Birraux, 2015 | No | Yes | Yes | Yes | Yes | Yes | Yes | Yes |
| Boia, 2014 | Yes | Yes | Yes | Yes | Yes | Yes | Yes | No |
| Braga, 2011 | No | No | Yes | Yes | Yes | Yes | Yes | Yes |
| Chowdhury, 2018 | Yes | Yes | Yes | Yes | Yes | No | No | Yes |
| CorrêaLeite, 2014 | No | No | Yes | Yes | Yes | Yes | Yes | Yes |
| Deshpande, 2020 | No | No | Yes | Yes | Yes | Yes | Yes | Yes |
| Elsawy, 2012 | No | Yes | Yes | Yes | Yes | Yes | Yes | Yes |
| Fukui, 2012 | No | Yes | Yes | Yes | Yes | Yes | No | No |
| Garge, 2014 | No | No | Yes | Yes | Yes | No | Yes | No |
| Gozar, 2014 | Yes | Yes | Yes | Yes | Yes | Yes | Yes | Yes |
| Gupta, 2018 | No | Yes | Yes | No | Yes | No | No | Yes |
| Joshi, 2007 | No | Yes | Yes | Yes | Yes | No | Yes | Yes |
| Kamble, 2015 | No | No | Yes | Yes | Yes | No | No | No |
| Keir, 2009 | Yes | Yes | Yes | Yes | Yes | Yes | Yes | Yes |
| Kendrick, 2021 | No | No | Yes | Yes | Yes | No | Yes | Yes |
| Kumar, 2015 | No | Yes | Yes | Yes | Yes | Yes | No | Yes |
| Kundal, 2013 | No | Yes | Yes | Yes | Yes | Yes | No | Yes |
| Levy, 2023 | No | Yes | Yes | Yes | No | Yes | No | Yes |
| Liu, 2010 | No | Yes | Yes | Yes | Yes | Yes | Yes | Yes |
| Macedo, 2009 | No | No | Yes | Yes | Yes | Yes | No | No |
| Macedo, 2015 | No | No | No | No | Yes | Yes | Yes | No |
| Macedo, 2022 | No | No | Yes | Yes | Yes | No | No | Yes |
| Matsumoto, 2012 | No | Yes | Yes | Yes | Yes | Yes | No | No |
| Matsumoto, 2016 | No | Yes | Yes | Yes | Yes | Yes | Yes | No |
| Mirshemirani, 2010 | Yes | Yes | Yes | Yes | Yes | Yes | Yes | No |
| NoumanAli, 2022 | No | No | Yes | Yes | No | No | No | No |
| Oyania, 2023 | No | Yes | Yes | Yes | Yes | No | Yes | Yes |
| Ozsu, 2013 | No | No | Yes | Yes | Yes | No | No | No |
| Parelkar, 2009 | No | No | Yes | Yes | Yes | No | No | Yes |
| Paula, 2015 | Yes | Yes | Yes | Yes | Yes | Yes | Yes | No |
| Rahayatri, 2021 | No | Yes | Yes | Yes | Yes | Yes | Yes | Yes |
| Samadi, 2021 | No | Yes | Yes | Yes | Yes | Yes | Yes | No |
| Sekhon, 2017 | No | No | Yes | Yes | Yes | Yes | No | No |
| Tran, 2011 | No | Yes | Yes | Yes | Yes | Yes | No | No |
| Tuna, 2019 | No | Yes | Yes | Yes | Yes | Yes | Yes | Yes |
| Vivier, 2011 | No | Yes | Yes | Yes | Yes | Yes | Yes | Yes |

*Case Series (Johanna Briggs Institute Quality Assessment Tool -* [*https://jbi.global/critical-appraisal-tools*](https://jbi.global/critical-appraisal-tools)*)*

|  | Were there clear criteria for inclusion in the case series? | Was the condition measured in a standard, reliable way for all participants included in the case series? | Were valid methods used for identification of the condition for all participants included in the case series? | Did the case series have consecutive inclusion of participants? | Did the case series have complete inclusion of participants? | Was there clear reporting of the demographics of the participants in the study? | Was there clear reporting of clinical information of the participants? | Were the outcomes or follow up results of cases clearly reported? | Was there clear reporting of the presenting site(s)/clinic(s) demographic information? | Was statistical analysis appropriate? |
| --- | --- | --- | --- | --- | --- | --- | --- | --- | --- | --- |
| Acimi, 2013 | Yes | Yes | Unclear | Yes | No | No | Yes | Yes | No | Not applicable |
| Acimi, 2018 | Yes | Yes | Unclear | Yes | Yes | No | Yes | Yes | No | Not applicable |
| Acimi, 2019 | Yes | Yes | Unclear | Yes | Yes | No | No | Yes | No | Not applicable |
| Akbiyik, 2010 | Yes | Unclear | Unclear | Yes | Yes | No | Yes | Yes | No | Not applicable |
| Bose, 2022 | Yes | Yes | Yes | Yes | Yes | Yes | Yes | Yes | No | Not applicable |
| Correya, 2021 | No | Unclear | Unclear | No | Unclear | No | Yes | Yes | No | Not applicable |
| Dehneh, 2022 | Yes | Yes | Unclear | Yes | Yes | Yes | Yes | Yes | No | Not applicable |
| Ferong, 2020 | Yes | Unclear | Unclear | Yes | Yes | No | Yes | Yes | No | Not applicable |
| Koncova, 2019 | Yes | Yes | Unclear | No | Unclear | Yes | Yes | Yes | No | Not applicable |
| Ozturk, 2007 | Yes | Unclear | Unclear | No | Unclear | No | Yes | No | No | Not applicable |
| Scarpa, 2019 | Yes | Unclear | Unclear | No | Unclear | No | Yes | Yes | No | Not applicable |
| Wester, 2012 | Yes | Unclear | Unclear | No | Unclear | No | Yes | Yes | No | Not applicable |
| Wolffenbuttel, 2019 | Yes | Unclear | Unclear | Yes | Unclear | No | Yes | Yes | No | Not applicable |

*Cohort Studies (Johanna Briggs Institute Quality Assessment Tool -* [*https://jbi.global/critical-appraisal-tools*](https://jbi.global/critical-appraisal-tools)*)*

|  | Were the two groups similar and recruited from the same population? | Were the exposures measured similarly to assign people to both exposed and unexposed groups? | Was the exposure measured in a valid and reliable way? | Were confounding factors identified? | Were strategies to deal with confounding factors stated? | Were the groups/participants free of the outcome at the start of the study (or at the moment of exposure)? | Were the outcomes measured in a valid and reliable way? | Was the follow up time reported and sufficient to be long enough for outcomes to occur? | Was follow up complete, and if not, were the reasons to loss to follow up described and explored? | Were strategies to address incomplete follow up utilized? | Was appropriate statistical analysis used? |
| --- | --- | --- | --- | --- | --- | --- | --- | --- | --- | --- | --- |
| Baskin, 2020 | No | Yes | Not applicable | No | No | Not applicable | No | Yes | No | No | Yes |
| Bernabe, 2018 | Not applicable | Not applicable | Not applicable | No | No | Not applicable | No | Yes | Unclear | Not applicable | Yes |
| Dangle, 2017 | Not applicable | Not applicable | Not applicable | Yes | No | Not applicable | Yes | Yes | Unclear | Not applicable | No |
| Elsayed, 2020 | No | Yes | Not applicable | Yes | No | Not applicable | Yes | Yes | Unclear | Not applicable | Yes |
| Erginel, 2023 | Yes | Yes | Not applicable | No | No | Not applicable | No | Yes | No | No | Yes |
| Fares, 2019 | Not applicable | Not applicable | Not applicable | No | No | Not applicable | No | Yes | Unclear | Not applicable | No |
| Fernandez, 2021 | Not applicable | Not applicable | Not applicable | No | No | Not applicable | No | Yes | Yes | Not applicable | No |
| Jesus, 2018 | Not applicable | Not applicable | Not applicable | No | No | Not applicable | No | Yes | No | No | No |
| Kirli, 2013 | Not applicable | Not applicable | Not applicable | Yes | No | Not applicable | No | Yes | No | No | No |
| Kudela, 2020 | Not applicable | Not applicable | Not applicable | Yes | No | Not applicable | No | Yes | Unclear | Not applicable | Yes |
| Matsui, 2011 | Not applicable | Not applicable | Not applicable | Yes | No | Not applicable | No | Yes | No | No | No |
| Nasir, 2019 | Not applicable | Not applicable | Not applicable | Yes | No | Not applicable | No | Yes | Unclear | Not applicable | Yes |
| Nokoff, 2017 | Unclear | Yes | Not applicable | Yes | No | Not applicable | No | Yes | No | No | Yes |
| Park, 2011 | Not applicable | Not applicable | Not applicable | No | No | Not applicable | No | Yes | Unclear | Not applicable | No |
| Podesta, 2008 | Not applicable | Not applicable | Not applicable | No | No | Not applicable | No | Yes | Unclear | Not applicable | No |
| Rehman, 2020 | Not applicable | Not applicable | Not applicable | Yes | No | Not applicable | No | Yes | Unclear | Not applicable | No |
| Roll, 2006 | Not applicable | Not applicable | Not applicable | No | No | Not applicable | No | Yes | Unclear | Not applicable | No |
| Savanelli, 2008 | Not applicable | Not applicable | Not applicable | No | No | Not applicable | No | Yes | Unclear | Not applicable | No |
| VanDerZwan, 2013 | Unclear | Yes | Not applicable | Yes | No | Not applicable | Yes | Yes | No | Yes | Yes |
