# Supplementary material for: Perspectives on conducting “sex-normalising” intersex surgeries conducted in infancy: A systematic review
Source: PLOS Glob Public Health. 2024 Aug 28;4(8):e0003568. doi: 10.1371/journal.pgph.0003568 (PMC11356455; doi:10.1371/journal.pgph.0003568)
Supplement: S3 Table — (DOCX) [file pgph.0003568.s005.docx]

|  |  |  |  |  |  |  |  |  |  |  |  |  |
| --- | --- | --- | --- | --- | --- | --- | --- | --- | --- | --- | --- | --- |
| **Study** | **Data extractors (date of extraction0** | **Country of first author** | **Congenital variations in sex characteristics^1^** | **“Sex-normalising” intervention conducted^2^** | **Study Design** | **Sample size (Relevant Sample Size^3^)** | **Participant Characteristics** | **Follow-up Time** | **Rationale for conduct coding** | **Rationale for conduct (verbatim)** | **Rationale for timing coding** | **Rationale for timing (verbatim)** |
| Acimi 2013 | CLA (Septembeer 2023)  LM (September 2023)  LG (October 2023) | Algeria | Congenital adrenal hyperplasia | Vaginoplasty | Case series | 27 (18) | Patients with 46 XX, DSD aged 4-28 months. All patients had 21-hydroxylase deficiency, four with simple virilising form and fourteen with additional salt loss. Three patients grade II on Prader scale, five grade III and 10 grade IV and V. | 6-48 months | Perceived goal of aligning with assigned sex + Anatomical/Functional/Cosmetic | The objective of feminizing genitoplasty is to allow the child to have a cosmetic aspect of the external genitalia corresponding to gender, a fundamental factor in childhood gender and psychosexual development and separation of the vagina from the urethra with the aim of having a vaginal introitus in the normal perineal position. The neo-vagina must enable the patient to experience in adulthood a normal sexual life, a normal menstrual flow, and even be able to deliver naturally without problems | Perceived goal of aligning with assigned sex + Parental Desire + Anatomical/Functional/Cosmetic | The surgical correction should be performed as early as possible to allow for good development of the patient’s sexual identity […] Several factors support this reasoning: The birth of a child with ambiguous genitalia is a tragedy for the parents, and prolonging this suffering is not justified, the missing part of the vagina is not very important in infants, and early assignment of an appropriate sex of rearing is important for childhood gender and psychosexual development. |
| Acimi 2018 | CA (September 2023)  LM (September 2023)  LG (October 2023) | Algeria | 46,XX disorder of sex development, ovotesticular disorder of sex development | Cliteroplasty, vaginoplasty, feminising genitoplasty | Case series | 29 (29) | Patients with 46, XX DSD, two with 11-hydroxylase deficiency and twenty-six with 21-hydroxylase deficiency and one ovotesticular DSD between 3-47 months. Four patients had grade II on Prader scale, six grade III and eighteen grade IV and V. | 0.5-12 years | Anatomical/Functional/Cosmetic + Perceived goal of aligning with assigned sex | A voluminous glans is often considered ugly due to its disproportionate size compared to the surrounding genitalia, which may be a source of dissatisfaction to the parents and patients […] surgical correction must create the appearance of external genitalia that correspond to the gender. | Anatomical/Functional/Cosmetic | The circumflex arteries of the penis begin to develop, only after the fourth year. This anatomical finding is an argument for performing clitoroplasty at an early age to avoid any risk of intraoperative and postoperative bleeding. […] In patients with 46,XX DSD and some other forms of DSD, the female gender is proposed and surgical correction should be performed as early as possible. |
| Acimi 2019 | CA (September 2023)  LM (September 2023)  LG (October 2023) | Algeria | 46, XX disorder of sex development, Ovotesticular disorder of sex development | Vaginoplasty | Case series | 22 (22) | Patients with 46, XX DSD, two with 11-hydroxylase deficiency and nineteen with 21-hydroxylase and one ovotesticular DSD between 4-47 months. Eight patients had grade III on Prader scale, fourteen had grade IV and V. | 4 months – 10 years | Perceived goal of aligning with assigned sex | In patients with 46, XX DSD and some other types of DSD, the female gender is proposed, and a surgical correction should be performed as early as possible to permit the development of a good gender identity in the patients. | Perceived goal of aligning with assigned sex | A surgical correction should be performed as early as possible to permit the development of a good gender identity in the patients. |
| Agarwal 2016 | CA (September 2023)  LM (September 2023)  LG (October 2023) | India | Mixed gonadal dysgenesis | Laparoscopic gonadectomy with vaginoplasty and clitoral reduction | Case study | 1 (1) | 7-year-old child with abnormal external genitalia, ovotesticular DSD and 46, XY/45, XO karyotype. | 2 years | Perceived goal of aligning with assigned sex | The patient was reared as a girl child because of external genitalia looking like a female. After the operation the same situation was maintained after excising the enlarged clitoris and the child has the potential to become a well-adjusted functional member of the society. | No rationale given | n/a |
| Agzamkhodjayev 2022 | CA (September 2023)  LM (September 2023)  LG (October 2023) | Uzbekistan | Diphallia | Left partial penectomy | Case study | 1 (1) | 7-year-old child with abnormal genitalia | 2 months | No rationale given | n/a | No rationale given | n/a |
| Akbiyik 2010 | LM (January 2024)  LG (January 2024) | Türkiye | Congenital adrenal hyperplasia | Feminising genitoplasty | Case series | 41 (41) | Patients aged 1-10 years with 21-hydroxylase deficiency | 1 month – 7.5 years | No rationale given | n/a | Perceived goal of aligning with assigned sex + Parental Desire | Surgery in infancy should be aimed principally at creating the appearance of normal external female genitalia, to alleviate parental distress and to avoid the potential psychological squeal of incorrect virilization in girls with CAH […] We now believe that it may be prudent to defer definitive vaginoplasty until puberty. An advantage of deferring vaginoplasty until puberty is the availability of supple, genital skin which provides more robust and amenable flap formation than the genital and introital skin of infants. |
| Attia 2023 | CA (September 2023)  LM (September 2023)  LG (October 2023) | Saudi Arabia | Ovotesticular disorder of sex development | Reconstructive vaginoplasty | Case study | 1 (1) | 2-month-old baby with abnormal external genitalia and labioscrotal folds | Not reported | Parental Desire | The parents and the team decided to raise the baby as a female. Therefore, the child had reconstructive vaginoplasty. | No rationale given | n/a |
| Baskin 2020 | CA (September 2023)  LM (September 2023)  LG (November 2023) | United States of America | Congenital adrenal hyperplasia, ovotesticular syndrome, mixed gonadal dysgenesis, partial androgen insensitivity syndrome | Feminising genitoplasty | Cohort | 57 (50) | Patients with 46, XX DSD due to CAH, partial androgen insensitivity syndrome, mixed gonadal dysgenesis and ovotesticular syndrome | 12 months | Anatomical/Functional/Cosmetic | Achieving "typical" female appearance and function in patients with moderate to severe genital atypia | No rationale given | n/a |
| Bernabé 2018 | CA (September 2023)  LM (September 2023)  LG (November 2023) | United States of America | Gonadal dysgenesis, partial androgen insensitivity syndrome, severe hypospadias and microphallus, congenital adrenal hyperplasia, chromosome mosaicism | Masculinising and feminising genitoplasty | Cohort | 27 (27) | Ten 46,XY patients with gonadal dysgenesis, partial androgen insensitivity syndrome, testosterone biosynthetic defect, severe hypospadias and microphallus. Sixteen 46, XY patients with CAH. One child with sex chromosome mosaicism (45,X/46,XY) | 12 months | No rationale given | n/a | No rationale given | n/a |
| Birraux 2015 | CA (September 2023)  LM (September 2023)  LG (October 2023) | Switzerland | Congenital adrenal hyperplasia | Feminising genitoplasty | Case study | 1 (1) | 3-year-old child with genital ambiguity, severe virilisation, Prader V stage and 46, XX karyotype | 2 months | No rationale given | n/a | No rationale given | n/a |
| Boia 2014 | CA (September 2023)  LM (September 2023)  LG (October 2023) | Romania | Antley-Bixler Syndrome | Combined perineal and transabdominal surgical intervention for ambiguous genitalia | Case study | 1 (1) | 3-year-old child with facial dimorphism, forearm and elbow malformations and ambiguous genitalia | 6 months | No rationale given | n/a | No rationale given | n/a |
| Bose 2022 | CA (September 2023)  LM (September 2023)  LG (November 2023) | India | 5-alpha reductase deficiency | Single stage urethroplasty, bilateral orchidopexy, correction of penoscrotal transposition, urethrocutaneous fistula closure, chordee correction, excision of UGS remanent, partial urethral tubularisation, excision of urogenital sinus reminant, release of right obstructed inguinal hernia | Case series | 12 (10) | 5-alpha reductase deficiency cases managed at a paediatric gender clinic ranging from 3 days-14 years old | 4-10 years | No rationale given | n/a | Perceived goal of aligning with assigned sex + Anatomical/Functional/Cosmetic + Parental Desire | The younger the child at gender assignment, the better is the adjustment of of the child/family to provide support for the management decision […] Late diagnosis leads to female sex of rearing, feminizing surgery and gonadectomy in childhood or incongruous pubertal virilization in those with retained gonads. Subsequent pharmacological induction of puberty and infertility culminates in poor outcome in adolescence and adulthood […] in the sociocultural context we practice in, the parents/caretakers are anxious to complete the procedures in early childhood before schooling and peer interactions. |
| Braga 2011 | LM (September 2023)  LG (October 2023) | Canada | Persistent cloaca | Posterior sagittal anorectoplasty and total urogenital sinus mobilisation | Case study | 1 (1) | 34-week gestation new-born with XX karyotype presenting with an enlarged clitoris, bilateral non-palpable gonads, and a single perineal orifice at 10 months | 6 months | Anatomical/Functional/Cosmetic | Allowed a more natural looking, and possibly functioning, vaginal introitus, improving the final cosmetic result […] Creating more normal-looking female external genitalia | No rationale given | n/a |
| Chowdhury 2018 | LM (September 2023)  LG (October 2023) | Bangladesh | Ambiguous genitalia | Orchiopexy, separation and closure of vaginal orifice, chordee correction, urethroplasty, scrotal reconstruction | Case study | 1 (1) | 8-months-old child presenting with enlarged clitoris, bifid scrotum and separate presence of urethral and vaginal orifices | Lost to follow-up | Perceived goal of aligning with assigned sex | To convert her into male sex | No rationale given | n/a |
| CorrêaLeite 2014 | LM (September 2023)  LG (October 2023) | Brazil | Diphallia | Penectomy | Case study | 1 (1) | 2-year-old boy with 46, XY karyotype presenting with two separate penises | 2 years | No rationale given | n/a | No rationale given | n/a |
| Correya 2021 | LM (September 2023)  LG (October 2023) | India | Gonadal dysgenesis | Hypospadias correction | Case series | 2 (2) | 6-month-old presenting with penoscrotal hypospadias, micropenis and right non-palpable testis | No follow-up | No rationale given | n/a | No rationale given | n/a |
| Dangle 2017 | LM (September 2023)  LG (December 2023) | United States of America | Congenital adrenal hyperplasia | Genitourinary reconstructive surgery | Cohort | 26 (26) | Patients with CAH aged 5-87 months old, with all but two diagnosed with classical CAH, with the other two diagnosed with non-classical | 4.5-142 months | Anatomical/Functional/Cosmetic | Favourable cosmetic and functional outcomes | Anatomical/Functional/Cosmetic + Perceived goal of aligning with assigned sex | Proponents of early repair support it on the basis of maternal estrogen effect, minimizing parenteral stress, better compliance, and no recollection of surgery later in adulthood. Others propose delayed intervention during adolescent age or beyond due to additional surgical interventions required following childhood reconstruction |
| Dehneh 2022 | LM (September 2023)  LG (November 2023) | Syrian Arab Republic | Congenital adrenal hyperplasia | Reconstructive surgery | Case series | 5 (4) | 46, XX Infant with male-appearing external genitalia and untraceable testes, 46, XX 5-year-old with ambiguous genitalia with adrenal hypertrophy and vagina and 46, XX infant with ambiguous genitalia | Not reported | Perceived goal of aligning with assigned sex + Anatomical/Functional/Cosmetic | Reconstructive surgery […] could offer psychological relief, by resolving the sexual ambiguity of the genitalia, and may facilitate sexual intercourse, although it may enhance the feeling of being different | Belief of Best Practice | Early surgical treatment, rather than delayed or staged approaches for 46, XX CAH patients with specific degrees of genital virilization, has been included in guidelines for the Development of Comprehensive Care Centers for Congenital Adrenal Hyperplasia. |
| Deshpande 2020 | LM (September 2023)  LG (October 2023) | India | Diphallia | Amputation of the left phallus | Case report | 1 (1) | 2-year-old with two well-formed penises | 10 months | No rationale given | n/a | No rationale given | n/a |
| Elsawy 2012 | LM (September 2023)  LG (October 2023) | Kuwait | Diphallia | Surgical reconstruction | Case study | 1 (1) | 37-day-old with duplicated penis | 1 year | No rationale given | n/a | No rationale given | n/a |
| Elsayed 2020 | LM (September 2023)  LG (November 2023) | Egypt | Congenital adrenal hyperplasia | Urogenital sinus mobilisation and nerve-sparing cliteroplasty | Cohort | 61 (35) | 61 children who underwent feminising genitoplasty, 35 of whom had this conducted before 2 years of age (aged between 3 days-10 months), and 26 after. | 1-12 years | Anatomical/Functional/Cosmetic | Correct the external genitalia and separate the genital from urinary tract with correction of the external genitalia achieving a desirable cosmetic and functional outcome | No rationale given | n/a |
| Erginel 2023 | LM (September 2023)  LG (December 2023) | Türkiye | Congenital adrenal hyperplasia | Cliteroplasty and vaginoplasty | Cohort | 14 (14) | 14 patients aged 10-96 months who underwent feminising genitoplasty, 7 of whom were under the age of 2 | 3-18 years | Anatomical/Functional/Cosmetic | To create a female-like external genitalia, including an introitus that will allow intercourse, ensure menstrual flow […] to promote the female gender and to avoid psychological stress on the parents and girls | Perceived goal of aligning with assigned sex | Before two years of age, children are psychosocially neutral; it is important to perform the operations before this age because the child is not likely to remember the surgery. The reconstructive process would have been completed before sexual identity was established […] performed infant genital surgeries promote the female gender and to avoid psychological stress on the parents and girls |
| Fares 2019 | LM (September 2023)  LG (December 2023) | Egypt | Congenital adrenal hyperplasia | Surgical management of high urogenital sinus | Cohort | 7 (7) | Patients aged between 12 months-5 years with a preconfluence urethra of < 15mm | 12-18 months | Anatomical/Functional/Cosmetic | Aiming to produce a feminine appearance, preserving the delicate genital nerve supply | Perceived goal of aligning with assigned sex | Patients classified according to Prader’s classification (from III to V degrees), would arguably need a form of clitoroplasty. One of the established strategies is a single-stage early feminizing surgery, aiming to produce a feminine appearance, preserving the delicate genital nerve supply […] aiming to confer an early physical appearance consistent with the female gender of rearing, and to cause less psychological stigmatization than with delayed surgery. |
| Fernandez 2021 | LM (September 2023)  LG (November 2023) | Colombia | Congenital adrenal hyperplasia | Complete corporeal preservation cliteroplasty, vaginoplasty | Cohort | 4 (4) | Patients with mean age of 18.5 months and a molecular confirmed diagnosis of CAH | 1 year | Anatomical/Functional/Cosmetic + Belief of Best Practice | Restore female aspect of genitalia while preserving dorsal neurovascular bundle but not at the expense of not preserving erectile tissue […] In the last consensus statement on the management of DSD, it was suggested to perform early surgery and UGS repair in girls with severe virilization | Perceived goal of aligning with assigned sex + Parental Desire + Anatomical/Functional/Cosmetic + Belief of Best Practice | Most families opt for early surgery, considering the positive implications for children’s psychosocial development, relieving parents distress and restoring “normal” external genital configuration […] The 4th World Congress of the International Society of Hypospadias and Disorders of Sex Development Surgery and the American Academy of Pediatrics, suggested performing the surgery before 2 years of age. |
| Ferong 2020 | LM (September 2023)  LG (December 2023) | Belgium | 45,X/46,XY disorder of sex development | Resection of gonadal structure | Case series | 6 (1) | New-born with diagnosis of 45,X/46,XY DSD referred for management of penoscrotal hypospadias and non-palpable testis | 6 months | No rationale given | n/a | No rationale given | n/a |
| Fukui 2012 | LM (September 2023)  LG (November 2023) | Japan | Ovotesticular disorder of sex development | Herniorrhaphy and removal of right gonad, uterus and fallopian tubes with one-stage hypospadias repair | Case study | 1 (1) | 1-year-old child presenting with hypospadias, right undescended testes and asymmetric external genitalia | Not reported | No rationale given | n/a | No rationale given | n/a |
| Garge 2014 | LM (September 2023)  LG (November 2023) | India | Herlyn-Weber-Wunderlich Syndrome | Septoplasty to create a single vaginal orifice | Case study | 1 (1) | 10-year-old child presenting with well-defined mass in suprapubic region with vulvar mass and a normal vaginal and urethral orifice | 2 months | No rationale given | n/a | No rationale given | n/a |
| Gozar 2014 | LM (September 2023)  LG (November 2023) | Romania | Congenital adrenal hyperplasia | Cliteroplasty, reconstruction of labia minora, creation of neovulva and vaginoplasty | Case study | 1 (1) | 3-year-old child presenting with history of classic CAH, with external genital pigmentation, cliteromegaly and labioscrotal fusion | 4 years | Anatomical/Functional/Cosmetic + Perceived goal of aligning with assigned sex | To help normal psychosexual development and creation of a functional vagina to allow menstruation and sexual activity | Belief of Best Practice | The Endocrine Society suggests that in patients with a low vaginal confluence, complete repair, including vaginoplasty, perineal reconstruction, and clitoroplasty (if necessary), can be done at an early age |
| Gupta 2018 | LM (September 2023)  LG (October 2023) | India | Persistent Müllerian duct syndrome | Left herniotomy with bilateral trans-septal orchiodopexy | Case study | 1 (1) | 1-year-old with swelling in left inguinal region and absent right testis since birth | 2 days | No rationale given | n/a | No rationale given | n/a |
| Jesus 2018 | LM (September 2023)  LG (November 2023) | Brazil | Genital ambiguity | Feminising genitoplasty with total urogenital sinus mobilisation | Cohort | 8 (7) | Six patients with CAH and two with mixed gonadal dysgenesis, with a mean age of surgery of 51 months | 3-56 months | Belief of Best Practice + Anatomical/Functional/Cosmetic | According to the Chicago Consensus 2006, feminizing genitoplasty, when indicated, should be performed in the most virilized cases (Prader III to V) and should be performed in specialized centers, with the focus being on future sexual function, not just cosmetic appearance […] this technique, when indicated, is feasible and effective in achieving the objectives of this type of surgery; i.e. adequate separation between the vagina and urethra to achieve high urethrovaginal confluence, a well-positioned clitoris and a satisfactory external appearance of the vagina. | No rationale given | n/a |
| Joshi 2007 | LM (September 2023)  LG (November 2023) | United Kingdom of Great Britain and Northern Ireland | Mixed gonadal dysgenesis | Laparoscopic excision of Mullerian structures | Case study | 1 (1) | 2-day old neonate presenting with proximal hypospadias and a non-palpable right gonad | 2 days | Perceived goal of aligning with assigned sex | As gender assignment had already been agreed, we undertook an early excision of the Mullerian structures | Anatomical/Functional/Cosmetic | Considering the size of the hydrometrocolpos, the potential risk of infection, and the pressure effects over adjacent structures, we undertook an early surgical intervention […] The failure to recognize and promptly manage a hydrocolpos can lead to pyocolpos, vaginal perforation, persistent bilateral hydronephrosis, megaureters, recurrent urinary tract infections, persistent acidosis, and a failure to thrive. The size of the hydrometrocolpos in this patient prompted us to decide on an early surgical intervention |
| Kamble 2015 | LM (September 2023)  LG (November 2023) | India | Transverse testicular ectopia with persistent Müllerian duct syndrome | Laparoscopic correction with division and removal of uterus | Case study | 1 (1) | 4-month-old with non-palpable right testis and left sided inguinal hernia | 6 months | No rationale given | n/a | No rationale given | n/a |
| Keir 2009 | LM (September 2023)  LG (October 2023) | United Kingdom of Great Britain and Northern Ireland | Congenital adrenal hyperplasia | Removal of Mullerian structures | Case study | 1 (1) | 5-year-old with micropenis, penile hypospadias and impalpable testes | Not reported | Parental Desire | The parents, particularly the father, expressed a strong desire for removal of the Mullerian organs. Given the recurrent abdominal pain and the presence of haematocolpos, there may have been a clinical basis for surgery, but initially the clinical team discouraged it, explaining that at this young age the procedure is permanent and cannot be reversed. However, as the family was returning to their country of origin where this procedure may have been performed by less experienced surgeons, the clinical team proceeded to surgery to remove the patient’s Mullerian organs. | Access | Ultimately, because the patient and family were only temporary visitors to the UK, in the interests of the child we decided to perform the surgery in our expert unit rather than risk having it performed at a non-specialist facility in the country of origin |
| Kendrick 2021 | LM (September 2023)  LG (November 2023) | Australia | Diphallia | Excision of the right phallus | Case study | 1 (1) | Neonate presenting with penile duplication | Not reported | No rationale given | n/a | No rationale given | n/a |
| Kirli 2013 | LM (September 2023)  LG (December 2023) | Türkiye | Congenital adrenal hyperplasia | Hysteroectomy with bilateral salphingo-oophorectomy and vaginectomy, chordee release, urethra repair, surrenalectomy, mastectomy, fistula repair | Cohort | 11 (11) | Patients ranging from 5 days-10 years, presenting with nonpalpable gonads, hyperpigmentation, jaundice and electrolyte imbalance, all with non-palpable gonads | Not reported | Perceived goal of aligning with assigned sex + Anatomical/Functional/Cosmetic | Because genital appearance is one of the main factors of normal sexual development, successful genital surgery is the important step of treatment […] In this situation, discordance between genetic gender and phenotypic sexual characteristics faces the gender assignment team including paediatric surgeon to a conflict. Unfortunately, paediatric surgeon is obligated to plan corrective surgery of masculinising genitoplasty […] Aim of the treatment is to provide normal physical and psychosocial development with protection of fertility of the patient whenever possible […] In a case with delayed diagnosis of 46XX virilising CAH, implementation of male reconstructive surgery can provide satisfactory male gender identity and heterosexual orientation if phenotype and chosen identity is male. | Perceived goal of aligning with assigned sex + Parental Desire + Anatomical/Functional/Cosmetic | Early diagnosis and management of intersex disorder is essential to provide normal metabolic, physical and psychosocial development […] In our opinion, surgical procedures should be performed as soon as the gender is assigned for the comfort of child and family. |
| Kocova 2019 | LM (September 2023)  LG (December 2023) | North Macedonia | 5-alpha reductase deficiency | Orchidectomy and cliteroplasty | Case study | 2 (1) | 20-month-year old with rugged labia resembling scrota, phallus and a perinaeal opening | 20 years | No rationale given | n/a | Perceived goal of aligning with assigned sex | Timing of putative sex reversal is important, for example, 5-ARD deficiency has been confirmed in female athletes excelling in athletics, thus complicating their sports life […] The recent guidelines should be followed in 46,XY under virilized babies, and leave the possibility for sex reversal for later in life, if needed. |
| Kudela 2020 | LM (September 2023)  LG (December 2023) | Poland | Congenital adrenal hyperplasia | Feminising genitoplasty | Cohort | 31 (31) | Group with mean age of 19 months consisting of 29 with 21-hydroxylase deficiency and 2 with 11-beta-hydroxylase deficiency, 7 having Prader grade III external virilisation, 21 with grade IV and 3 with grade V | 12 months-15 years | Perceived goal of aligning with assigned sex + Parental Desire + Anatomical/Functional/Cosmetic | Female gender identity can be expected in a person with 46,XX karyotype and CAH, therefore early feminizing genitoplasty in these cases seems to be justified. Surgical correction of even very severe virilization in female patients with CAH can restore female-pattern appearance of the genitalia […] All parents demanded early genital reconstructions despite the information from the multidisciplinary team about the option of postponing the reconstructive operation until the age of consent […] The purpose of the operation is to restore proper functional and female-looking anatomy of the genitalia. | Parental Desire + Belief of Best Practice | Female gender identity can be expected in a person with 46,XX karyotype and CAH, therefore early feminizing genitoplasty in these cases seems to be justified […] According to the current Endocrine Society guidelines in minimally virilized girls, observation or delayed surgery are preferred, however in severe virilized CAH females, early reconstruction is recommended […] Patients with CAH and 46,XX karyotype usually have no gender identity problems. The rare exceptions are lately diagnosed severely virilized cases who were assigned at birth and raised as male. Therefore, the majority of parents of 46,XX CAH patients desire early corrective surgery […] Similar to hypospadias, which is another much more common defect of the genitalia, it is probably better to perform the genital reconstruction between 6 to 18 months of age. This age is believed to be the best for corrective surgery based on psychological aspects […] Although there is no clear evidence in the literature showing that early surgery of 46,XX CAH is superior to late surgery, we believe that atypical genitalia including large clitorises may cause much psychological harm to patients and their families. |
| Kumar 2015 | LM (September 2023)  LG (November 2023) | India | True hermaphroditism | Total abdominal hysterectomy, bilateral salphingo-oophorectomy and colpectomy | Case study | 1 (1) | 3-year-old child admitted with ambiguous genitalia, with swellings in inguinal region, non-visible testis and scrotum and presence of enlarged clitoris | Not reported | Perceived goal of aligning with assigned sex | Child was made made after extirpation of female genitals […] If the patient is to be raised as female, all testicular and wolffian tissues should be removed | Anatomical/Functional/Cosmetic | If a male gender is assigned, as has been most common historically, all ovarian and Mullerian tissue should be removed. |
| Kundal 2013 | LM (September 2023)  LG (November 2023) | India | Diphallia | Penile amputation and phalloplasty | Case study | 1 (1) | 3-year-old presenting with two separate phalluses | Not reported | No rationale given | n/a | No rationale given | n/a |
| Levy 2023 | LM (September 2023)  LG (November 2023) | United States of America | 5-alpha reductase deficiency | Staged hypospadias repair | Case study | 1 (1) | Infant presenting with 2-cm genital tubercle with a single perineal opening and bifid labioscrotal folds | Not reported | No rationale given | n/a | No rationale given | n/a |
| Liu 2010 | LM (September 2023)  LG (November 2023) | China | Congenital adrenal hyperplasia | Cliteroplasty, reconstruction of labia minora | Case study | 1 (1) | 4-year-old child presenting with enlarged, hypertrophied clitoris | Not reported | Anatomical/Functional/Cosmetic + Perceived goal of aligning with assigned sex + Parental Desire | To restore the female’s external genital appearance and function […] Many reports emphasize the importance of reconstructing the clitoris to restore its physical appearance and its sensitivity to sexual stimuli […] The larger erectile clitoris can embarrass the parents and bring pain to the child, passively influencing development of the sexual psyche. Meanwhile, the absence of a vagina will not affect a small child. Sometimes, restoration of the vulva’s appearance is more important to the female pseudohermaphrodite girl than vaginal reconstruction. | Perceived goal of aligning with assigned sex + Anatomical/Functional/Cosmetic | Considering the deficiency and hypogenesis of the local tissue, surgical treatment was immediately considered due to the child’s mental development […] Several factors influence the timing of elective gender remodelling, including age-related aesthetics and surgical risks and benefits in relation to the psychosexual impact of the procedure during the various stages of development. Performing a genitoplasty before the age of 30 months seems to be important because awareness of sexual identity begins at that time […] The repair of genital malformation as early as possible will help patients to achieve a psychologically healthy body image |
| Macedo 2009 | LM (September 2023)  LG (November 2023) | Brazil | Transverse testicular ectopia and persistent Müllerian duct syndrome | Excision of Mullerian remnant | Case study | 1 (1) | 1-year-od child presenting with bilateral cryptorchidism with a uterus | Not reported | No rationale given | n/a | No rationale given | n/a |
| Macedo 2015 | LM (October 2023)  LG (December 2023) | Brazil | Congenital adrenal hyperplasia | Total urogenital mobilisation | Case study | 1 (1) | 9-month-old child presenting with Prader III virilisation of external genitalia and 46, XX karyotype | Not reported | No rationale given | n/a | No rationale given | n/a |
| Macedo 2022 | LM (October 2023)  LG (November 2023) | Brazil | Diphallia | Removal of left penile | Case study | 1 (1) | 2-year-old child with penile duplication | Not reported | Anatomical/Functional/Cosmetic | The goal of attaining satisfactory functional and cosmetic results. | No rationale given | n/a |
| Matsui 2011 | LM (October 2023)  LG (December 2023) | Japan | Ovotesticular disorder of sex development | Cliteroplasty, vaginoplasty, urethroplasty, scrotoplasty and uterocolpectomy | Cohort | 8 (8) | Children presenting at mean age of 2.4 months, with ambiguous genitalia, isolated clitoromegaly, perineal hypospadias and cryptorchidism | 3-16 years | Perceived goal of aligning with assigned sex | Surgery is generally necessary after gender assignment. This includes removal of gonads and internal ducts inappropriate to the sex of rearing, and genitoplasty to construct the appropriate external appearance. | Parental Desire + Perceived goal of aligning with assigned sex | Despite informing families of all treatment options available, they have often desired early gonadal surgery and genitoplasty. We believe early operations improve the attachment between a child and parents and benefit the development of gender identity in childhood. |
| Matsumoto 2012 | LM (October 2023)  LG (December 2023) | Japan | Ovotesticular disorder of sex development | Resection of ovarian segment | Case study | 1 (1) | 7-year-old child with ascent of scrotal contents, 46, XX karyotype and history of Mullerian remnant resection | 7 years | No rationale given | n/a | No rationale given | n/a |
| Matsumoto 2016 | LM (October 2023)  LG (November 2023) | Japan | Diphallia | Surgical correction of penis and urethra | Case study | 1 (1) | 9-month-old child presenting with two separate phalluses bifid scrotum and hypospadic urethral meatus | 5 years | Anatomical/Functional/Cosmetic | To achieve good functional and cosmetic outcomes | No rationale given | n/a |
| Mirshemirani 2010 | LM (October 2023)  LG (November 2023) | Iran (Islamic Republic of) | Diphallia | Reimplantation and resection of the left phallus | Case study | 1 (1) | 2-day-old chid presenting with with duplicated penis and proximal hypospadias on left penile | 4 years | No rationale given | n/a | No rationale given | n/a |
| Nasir 2019 | LM (October 2023)  LG (December 2023) | Nigeria | Disorders of sexual development | Urethroplasty, penis straightening, scrotoplasty, orchidopexy, feminising genitoplasty, gonadectomy | Cohort | 15 (14) | Fifteen children presenting with DSDs with a median age of 20 months. Ten presenting with ambiguous genitalia and five presenting with hypospadias | 2-26 months | Perceived goal of aligning with assigned sex | Accurately and appropriately assign sex | Perceived goal of aligning with assigned sex | A delay in making gender assignment or reassignment of the wrong gender is often fraught with emotional trauma and psychosocial issue […] it is generally agreed that the diagnosis of DSD should be promptly established after delivery and preferably before discharge so that an early sex of rearing can be assigned to an affected child and treatment can be planned |
| Nokoff 2017 | LM (October 2023)  LG (December 2023) | United States of America | Disorders of sex development | Feminising and masculinising genitoplasty | Cohort | 37 (35) | Children presenting with DSDs ranging from 5.4-29.7 months old. Twenty children had a 46, XX karyotype, fifteen had 46, XY and two had chromosome mosaicism | 6 months | Belief of Best Practice | It is recommended that genital surgery for a child raised as a female only be considered in cases of severe virilization (Prader 3–5) and that surgery of the clitoris not be performed for reasons of cosmetic appearance alone | Belief of Best Practice + Anatomical/Functional/Cosmetic | It is recommended that clitoral and perineal reconstruction be considered in infancy and those with a low vaginal confluence undergo vaginoplasty at an early age; the appropriate timing is less certain for those with a higher vaginal confluence […] Hypospadias repair is more successful if performed in pediatric rather than adult patients |
| NoumanAli 2022 | LM (October 2023)  LG (November 2023) | Pakistan | Diphallia | Distal penis resection | Case study | 1 (1) | 10-year-old presenting with double penis since childhood | Not reported | Anatomical/Functional/Cosmetic | To achieve normal contour of the genitalia | No rationale given | n/a |
| Oyania 2023 | LM (October 2023)  LG (November 2023) | Uganda | Mayer-Rokitansky-Küster-Hauser Syndrome | Vaginal reconstruction | Case study | 1 (1) | 3-year-old presenting with sigmoid colostomy and unrecognised MRKH syndrome | Not reported | Anatomical/Functional/Cosmetic | Creating a new cavity and replacing the vagina with a mucous membrane lined canal such as a segment of bowel | No rationale given | n/a |
| Ozsu 2013 | LM (October 2023)  LG (November 2023) | Turkey | Ovotesticular disorder of sexual development | Left gonadectomy and hypospadias repair | Case study | 1 (1) | 5-month old presenting with hypospadias, cryptorchidism and micropenis, with ovarian tissue, fallopian tubes and uterine remnants in left inguinal canal | 11 years | No rationale given | n/a | No rationale given | n/a |
| Ozturk 2007 | LM (October 2023)  LG (December 2023) | Türkiye | Persistent Müllerian Duct Syndrome with transverse testicular ectopia | Hysterectomy with resection of underdeveloped fallopian tubes | Case series | 2 (2) | 8-month-old child presenting with right incarcerated inguinal hernia and an empty left hemiscrotum | Not reported | No rationale given | n/a | No rationale given | n/a |
| Parelkar 2009 | LM (October 2023)  LG (December 2023) | India | Persistent Müllerian duct syndrome | Laparoscopic orchidopexy | Case study | 1 (1) | 10-month old infant presenting with left inguinal hernia and bilateral nonpalpable gonads | 6 months | Anatomical/Functional/Cosmetic | Placement of well-vascularized testes in the scrotum | No rationale given | n/a |
| Park 2011 | LM (October 2023)  LG (December 2023) | Republic of Korea | Genital ambiguity with high vaginal confluence. | Feminising genitoplasty, total urogenital mobilisation | Cohort | 10 (7) | Patients with median age at time of surgery of 21 months, seven with CAH, one with mixed gonadal dysgenesis, one with partial androgen insensitivity and one with 5-alpha reductase deficiency syndrome | 3-12 years | Anatomical/Functional/Cosmetic | Provide a normal cosmetic appearance without sacrificing sensation or vascularity of the glans, due to the importance of the clitoris in female sexual response and in achieving orgasm. | No rationale given | n/a |
| Paula 2015 | LM (October 2023)  LG (November 2023) | Brazil | Ovotesticular disorder of sex development | Left gonadectomy, removal of Mullerian structures and urethroplasty | Case study | 1 (1) | 2-month-old child with Prader stage III virilisation of external genitalia | 5 years | No rationale given | n/a | No rationale given | n/a |
| Podesta 2008 | LM (October 2023)  LG (December 2023) | Argentina | Congenital adrenal hyperplasia, partial androgen insensitivity, mixed gonadal dysgenesis | Feminising genital reconstruction | Cohort | 12 (12) | Patients ranging from 0.4-5.3 years old presenting with high entrance of vagina into the urethra | 3-12 years | Anatomical/Functional/Cosmetic | Give the urinary tract and genital apparatus as normal an anatomical state as possible, while also providing physiologic functions […] producing an aesthetically pleasing feminine genital appearance | No rationale given | n/a |
| Rahayatri 2021 | LM (October 2023)  LG (November 2023) | Indonesia | Mayer-Rokitansky-Küster-Hauser | Vaginal reconstruction | Case study | 1 (1) | 15-month-old presenting with transverse colostomy and clitoromegaly | 2 weeks | Perceived goal of aligning with assigned sex | Reduce the impact that this condition may have on physiological processes in the future. | No rationale given | n/a |
| Rehman 2020 | LM (October 2023)  LG (December 2023) | Pakistan | Congenital adrenal hyperplasia | Feminising genitolplasty | Cohort | 32 (32) | Patients aged 6-18 months, 3 with Prader Scale grade I external virilisation, 6 with grade II, 13 with grade III and 10 with grade IV | 3 weeks | Anatomical/Functional/Cosmetic + Perceived goal of aligning with assigned sex | As the children retain the potential for normal sexual activity and fertility in classical CAH, the treatment is directed to attain anatomical and psychological female gender and for this, children undergo feminizing genitoplasty after detailed diagnostic assessment and counselling | No rationale given | n/a |
| Roll 2006 | LM (October 2023)  LG (December 2023) | Germany | Congenital adrenal hyperplasia | Complete one-stage genital reconstruction, cliteroplasty, | Cohort | 19 (19) | Patients aged 1-7-years-old, seventeen with salt-wasting CAH and one with simple virilisation | 6 months-32 years | Anatomical/Functional/Cosmetic | A normal looking sensate clitoris, an adequately sized and appropriately situated vagina and a good functional outcome are the main goals of feminising genital reconstructive surgery. | Perceived goal of aligning with assigned sex + Parental Desire | Will be performed as early as possible to prevent psychological disturbances in the children and the parents and to avoid delay of the vaginoplasty procedure |
| Samadi 2021 | LM (October 2023)  LG (November 2023) | United States of America | Diphallia | Phalloplasty | Case study | 1 (1) | 10-month-old with duplicated penis | 3 months | No rationale given | n/a | No rationale given | n/a |
| Savanelli 2008 | LM (October 2023)  LG (November 2023) | Italy | Congenital adrenal hyperplasia | Feminising genitoplasty | Cohort | 14 (14) | Patients aged between 6 months and 4 years, five of whom had a vaginal orifice next to the external urethral sphincter and nine presenting with a more distal form | 3 months-8 years | Belief of Best Practice + Anatomical/Functional/Cosmetic + Parental Desire + Perceived goal of aligning with assigned sex | Recent consensus statement on the management of intersex disorders suggests that cosmetic surgery in girls with severe virilization (Prader III to V) should be performed in the first year of life, when appropriate, in conjunction with common UGS repair […] many believe that early surgery is appropriate in these children for a better and malleable tissue. We privilege a full feminization in the first years of life in distal and high forms […] The excellent anatomical appearance of the vulva created at an early stage facilitates the parents’ acceptance and helps create a more normal parents-daughter attachment and family relationships. | Perceived goal of aligning with assigned sex + Anatomical/Functional/Cosmetic | There are also controversies regarding the need for clitoral reduction. However, leaving a grossly enlarged clitoris untouched during childhood underscores the psychological impact that this situation can cause to the untreated child […] many believe that early surgery is appropriate in these children for a better and malleable tissue |
| Scarpa 2019 | LM (October 2023)  LG (December 2023) | Italy | Ovotesticular differences of sex development | Orchiopexy and hypospadias repair, removal of testicular tissue | Case series | 3 (3) | Three patients aged 12-17 months presenting with genital ambiguity | 3 months | Perceived goal of aligning with assigned sex | Surgery is necessary after gender assignment and includes removal of gonads and internal ducts inappropriate to the sex of rearing and genitoplasty to construct the appropriate external appearance […] The fertility potential must be respected and a satisfactory result must be obtained […] we removed the female gonad because of its macroscopic aspect of streak gonad with a potential tendency to degeneration. Although the fertility potential in a male OT-DSD is doubtful, the future presence of estradiol in developing ovarian follicles could inhibit spermatogonia development in contralateral seminiferous tubules. There is no evidence that prophylactic removal of asymptomatic Müllerian remnants is required. If possible, we suggest leaving them in situ in male patients. In cases 2 and 3 we maintained the ovarian part of the ovotestis for preserving a potential fertility even if in case 3 the uterus was not found. The families of the last two cases were strongly oriented to a female sex of rearing. | Parental Desire | Families often ask for an early surgical solution to ensure child well-being within the family, the school and the society. |
| Sekhon 2017 | LM (October 2023)  LG (December 2023) | India | Persistent Müllerian Duct Syndrome | Excision of uterus, fallopian tubes and portion of vagina | Case study | 1 (1) | Two-month-old infant presenting with left irreducible inguinal hernia with right non-palpable undescended testis | Not reported | Anatomical/Functional/Cosmetic | Reducing the impact of various medical, social and psychological problems associated with this condition […] If PMDS is discovered as an unexpected finding while operating, biopsy of the gonadal tissue and Mullerian structures is warranted […] Parental counselling about the complex pathophysiology should be done, keeping in mind the psychosocial implications. Once the diagnosis of PMDS is confirmed, the surgical management consists of excision of Mullerian remnants with orchidopexy. | Anatomical/Functional/Cosmetic | Performing definitive surgery at an early age, thereby reducing the impact of various medical, social and psychological problems associated with this condition |
| Tran 2011 | LM (October 2023)  LG (December 2023) | United States of America | Ovotesticular disorder of sex development | Total abdominal hysterectomy, vaginectomy, bilateral gonadectomy, first-stage hypospadias repair | Case study | 1 (1) | 1-day-old infant presenting with fused bifid labioscrotal folds, impalpable gonads, small phallus with severe chordee and penoscrotal hypospadias | 19 months | Perceived goal of aligning with assigned sex | Removal of gonads and internal genitalia that oppose the assigned sex | No rationale given | n/a |
| Tuna 2019 | LM (October 2023)  LG (November 2023) | Portugal | Herlyn-Werner-Wunderlich Syndrome | Vaginal septotomy | Case study | 1 (1) | 4-day-old infant presenting with absent right kidney, pelvic cystic lesion and a bulging in the location of the vaginal introitus | 4 days | Anatomical/Functional/Cosmetic | Obstructive reproductive tract anomalies, such as HWWS, comprise a higher risk of hematosalpinx, endometriosis, and pelvic inflammatory disease, potentially threatening the fertility of these patients […] The preferred treatment consists in the excision of the obstructing vaginal septum. | Anatomical/Functional/Cosmetic | Early and accurate diagnosis and treatment are of the utmost importance to avoid complications and maintain the reproductive potential of the patients […] Our clinical case shows that prenatal suspicion and careful physical examination at birth allows early diagnosis and management of HWWS, which relates to better outcomes and avoidance of potential lifelong complications. |
| VanDerZwan 2013 | LM (October 2023)  LG (December 2023) | Netherlands | Disorders of sex development | Hypospadias correction, gonadectomy, orchiopexy, | Cohort | 14 (14) | Participants aged between 14-32-years-old who underwent surgical intervention between ages of 1-6-years-old with partial androgen insensitivity syndrome, mixed gonadal dysgenesis, ovotesticular DSD and undefined 46, XY DSD | Not reported | Anatomical/Functional/Cosmetic | The aim of masculinizing surgery in patients with disorders of sex development is to improve cosmesis and function of the external genitalia, to enable sexual intercourse and to avoid stigmatization | No rationale given | n/a |
| Vivier 2011 | LM (October 2023)  LG (December 2023) | France | Herlyn-Werner-Wunderlich syndrome | Trans-hymenal resection of vaginal septum | Case study | 1 (1) | 1-month-old with dilated left pelvic kidney, uterus didelphys and communication between the left hemi-uterus and a retrovesical fluid-filled pelvic structure | 1 month | Anatomical/Functional/Cosmetic | To prevent complications such as hemihaematocolpos and secondary endometriosis. | No rationale given | n/a |
| Wester 2012 | LM (October 2023)  LG (December 2023) | Spain, Finland, Sweden | Mayer-Rokitansky-Küster-Hauser (MRKH) syndrome | Sigmoid colovaginoplasty, vaginal pull-through | Case series | 7 (6) | Patients aged between 13 months-17.5 years presenting with MRKH syndrome | 1 month-1 year | No rationale given | n/a | Anatomical/Functional/Cosmetic | In patients with vaginal agenesis and anorectal malformation, the vagina is normally created at the time of anorectal reconstruction. The rationale for this is that the perineum and the tissue plane between the urinary tract and rectum is scarred after the anorectal reconstruction, making a secondary procedure more difficult. |
| Wolffenbuttel 2019 | LM (October 2023)  LG (December 2023) | Netherlands | Perineal hypospadias in disorders of sex development | Perineal hypospadias repair | Case series | 4 (4) | Patients aged between 6 monhts-5 years presenting with perineal hypospadias associated with a range of DSDs | 6 months-5 years | Anatomical/Functional/Cosmetic | The main rationale for adopting this conservative approach however is to minimize genital tissue removal in children with a not yet definite gender identity, which will certainly facilitate unforeseen future gender reassignment surgery | No rationale given | n/a |

**Notes:**

1. Terminology used for congenital variations in sex characteristics matches those reported by authors and does not necessarily reflect the current or correct terminology used in medical practice or in the intersex community.
2. Terminology used for surgical interventions matches those reported by authors and does not necessarily reflect the current or correct terminology used in medical practice or in the intersex community.
3. Relevant sample size is the number of patients within a cohort or case series that meet all inclusion criteria.
